# Fbxo45 facilitates the malignant progression of breast cancer by targeting Bim for ubiquitination and degradation

**DOI:** 10.1186/s12885-024-12382-8

**Published:** 2024-05-21

**Authors:** Mengmeng Zheng, Linfeng Wu, Rongyao Xiao, Jiaohao Cai, Weike Chen, Shurong Shen

**Affiliations:** Department of Oncology and Hematology, Wenzhou Hospital of Integrated Traditional Chinese and Western Medicine, Wenzhou, Zhejiang China

**Keywords:** Breast cancer, Fbxo45, Bim, Proliferation, Apoptosis

## Abstract

**Background:**

Breast cancer is one of the common malignancies in women. Evidence has demonstrated that FBXO45 plays a pivotal role in oncogenesis and progression. However, the role of FBXO45 in breast tumorigenesis remains elusive. Exploration of the regulatory mechanisms of FBXO45 in breast cancer development is pivotal for potential therapeutic interventions in patients with breast cancer.

**Methods:**

Hence, we used numerous approaches to explore the functions of FBXO45 and its underlaying mechanisms in breast cancer pathogenesis, including CCK-8 assay, EdU assay, colony formation analysis, apoptosis assay, RT-PCR, Western blotting, immunoprecipitation, ubiquitination assay, and cycloheximide chase assay.

**Results:**

We found that downregulation of FBXO45 inhibited cell proliferation, while upregulation of FBXO45 elevated cell proliferation in breast cancer. Silencing of FBXO45 induced cell apoptosis, whereas overexpression of FBXO45 inhibited cell apoptosis in breast cancer. Moreover, FBXO45 interacted with BIM and regulated its ubiquitination and degradation. Furthermore, knockdown of FBXO45 inhibited cell proliferation via regulation of BIM pathway. Notably, overexpression of FBXO45 facilitated tumor growth in mice. Strikingly, FBXO45 expression was associated with poor survival of breast cancer patients.

**Conclusion:**

Our study could provide the rational for targeting FBXO45 to obtain benefit for breast cancer patients. Altogether, modulating FBXO45/Bim axis could be a promising strategy for breast cancer therapy.

**Supplementary Information:**

The online version contains supplementary material available at 10.1186/s12885-024-12382-8.

## Background

Breast cancer is one of the most common malignancies in females. There are 2,262,419 new cases of breast cancer and 684,996 new deaths from breast cancer patients [1]. In the United States, there are estimated new breast cancer cases approximately 297,790 [2]. Triple-negative breast cancer (TNBC) has the absence of three receptors on the surface of tumor cells: ER (estrogen receptor), PR (progesterone receptor), and HER2 (human epidermal growth factor receptor 2) [3]. TNBC, accounting for approximately 15–20% of all breast cancer cases, represents a more aggressive and challenging type of breast cancer for treatment [4]. The mortality of female breast cancer has decreased due to mammography, earlier diagnose and improved therapy. Treatment modalities include lumpectomy, mastectomy, chemotherapy, radiation therapy, targeted therapy, hormone therapy and immunotherapy [5–7]. However, because of metastasis and drug resistance [8], breast cancer is the second greatest numbers of deaths after lung cancer in women in the United States [2]. Hence, it is essential to discover the mechanism of breast tumorigenesis and improve the prognosis of breast cancer.

It has been revealed that multiple factors are involved in developing breast cancer, such as age, family history, obesity and alcohol consumption. Genetic mutations, including BRCA1 and BRCA2, have been documented to participate in breast cancer occurrence [9]. It is known that F-box proteins are part of the Skp1-Cullin-F-box (SCF) complex, which regulates protein degradation [10, 11]. The SCF complex belongs to E3 ubiquitin ligase, which is responsible for tagging proteins with ubiquitin and leading to proteasome-dependent degradation [12, 13]. F-box proteins regulate numerous cellular processes, such as proliferation, DNA replication, autophagy, apoptosis, invasion, EMT (epithelial-mesenchymal transition) and metastasis [14]. Dysregulation of F-box proteins leads to the uncontrolled cell growth and causes the development of various diseases, including cancer [15]. Recently, F-box proteins have been reported to regulate breast cancer occurrence and progression [16, 17].

FBXO45 has been identified to regulate oncogenesis and tumor progression in various cancer types [18]. For example, FBXO45 has been reported to enhance the degradation of p73 in a proteasome-dependent manner [19]. Chen et al. reported that FBXO45 regulated cancer cell survival via targeting tumor-suppressor Par-4 (prostate apoptosis response-4) for degradation [20, 21]. Par-4 amino-terminal fragment (PAF) can bind with FBXO45 and abrogate Par-4-induced cancer cell apoptosis, which overcome therapy resistance in tumors [22]. FBXO45-MYCBP2 influenced the degradation of FBXW7 and governed mitotic cell fate [23]. CASP8AP2 (caspase-8-associated protein 2 or FLASH) blocked ZEB1 degradation by FBXO45 and SIAH1 ubiquitin ligases, leading to regulation of EMT [24]. IL-24 destabilized the stability of ZEB1 via increasing the expression of FBXO45 in human glioblastoma cells, contributing to inhibition of malignancy of glioblastoma [25]. Low expression of FBXO45 was reported to be correlated with gastric cancer progression and poor prognosis [26]. By a systematic analysis, FBXO45 was uncovered to be a potential target and prognostic biomarker for breast cancer [27]. However, the role of FBXO45 in breast oncogenesis remains unclear. Understanding the regulatory mechanisms of FBXO45 in breast tumorigenesis is important for potential therapeutic interventions in breast cancer. In this study, we used numerous approaches to determine the functions of FBXO45 and its underlaying mechanisms in breast cancer pathogenesis.

## Methods and materials

### Cell culture

Two breast cancer cell lines (MCF7 and MDA-MB-231) were bought from the Cell Bank of the Chinese Academy of Sciences (Shanghai, China). The cell lines were maintained in Dulbecco’s modified Eagle’s medium (DMEM; Gibco, Grand Island, NY, USA), which contains 10% fetal bovine serum (FBS) and 1% penicillin-streptomycin. All cells were cultured in an incubator at 37 °C with 5% CO_2_.

### Transfection

Breast cancer cells were seeded on 6-well plates. When cells were grown until 60–70% confluence, the various plasmids were transfected into the breast cancer cells by Lipofectamine 2000 (Invitrogen, USA). siRNAs targeting the open reading frames of FBXO45 were synthesized by GenePharma (Shanghai, China). The transfected cells were cultured for different times under the Results section.

### Quantitative real-time polymerase chain reaction

TRIzol reagent (Invitrogen) was used to isolate total RNA in transfected breast cancer cells. Then, cDNA was synthesized by reverse transcriptase kit based on the manufacturer’s protocol. Real-time polymerase chain reaction (RT-qPCR) was carried out by a SYBR® Green PCR Kit (Qiagen) to determine the expression of mRNAs in breast cancer cells. The relative expression levels of genes were calculated by the comparative Ct method (2 − ^ΔΔCt^). GAPDH (Glyceraldehyde 3-phosphate dehydrogenase) acted as an endogenous loading control. The primer sequences are: FBXO45 forward 5’-AGT GCC AAG GTT ATG TGG CAT TGC TG-3’; reverse 5’-AGA AAG CCA CTG TCA TCC GTC CAA A-3’; β-actin forward 5’-GGA GAT TAC TGC CCT GGC TCC TA-3’; reverse 5’-GAC TCA TCG TAC TCC TGC TTG CTG-3’.

### Western blotting

The transfected cells were lysed in RIPA buffer after they were washed for three times by PBS. Then, total proteins were harvested and further quantified using a BCA protein assay kit (Pierce, USA). Proteins were separated by electrophoresis in sodium dodecyl sulfate (SDS)-polyacrylamide gel. Proteins were further transferred onto polyvinylidene difluoride membranes. The membranes were blocked by 5% non-fat milk for 1 h at room temperature. The membranes were further incubated with the primary antibody at cold room overnight. After the membranes were washed for three times by TBST, they were incubated with secondary antibody for 1 h at room temperature. Proteins of interest were measured using a Bio-Rad Imaging System.

### Cell viability assay

Cell counting kit-8 (CCK8) assay was conducted to determine viability of breast cancer cells. Briefly, transfected breast cancer cells (5000 cells/well) were cultured on 96-well plates with full DMEM for different time points. Then, 10 µl CCK8 solution was added and incubated for 2 h at 37 °C. The absorbance was detected at 450 nm by a microplate reader.

### EdU (5-ethynyl-2′-deoxyuridine) assay

The transfected cells were seeded into 96-well plates for different time points. Then, the cells were treated with 100 µL medium containing EdU for 2 h at 37 °C. Cells were fixed with 4% paraformaldehyde for 30 min and incubated with 0.5% TritonX-100 for 10 min. Cells were stained with 100 µL Apollo reaction solution for 30 min. Subsequently, cells were stained by Hoechst for 30 min. A fluorescence microscope was used to take images.

### Colony formation assay

The transfected breast cancer cells were seeded in 6-well plates. After cells were cultured for two weeks, the colonies were observed and washed three times with PBS. The colonies were treated with 4% paraformaldehyde for 30 min. Subsequently, the colonies were stained with 1% crystal violet. The colonies were imaged.

### Apoptosis assay

The transfected breast cancer cells were seeded in 6-well plates. After 48 h, cells were harvested and washed three times with PBS. Then, cells were resuspended in 500 µL binding buffer. Cells were further stained by PI reagent and Annexin V-FITC (fluorescein isothiocyanate) for 20 min at room temperature. Apoptotic cells were detected by the FACS flow cytometer.

### Invasion assay

The transfected breast cancer cells were seeded in the upper chamber with serum-free medium. The lower chamber was filled with medium supplemented with 10% FBS. After 24 h, the invaded cells on the lower chamber surfaces were fixed in 4% paraformaldehyde and stained in Calcein AM. The stained cells were photographed and counted in five random fields.

### Immunoprecipitation

The transfected cells were washed with PBS for three times. Then, cells were lysed in the immunoprecipitation lysis buffer (25mM Tris-HCL pH 7.4, 1 mM EDTA, 5% glycerol, 1% NP-40, 150 mM NaCl, 1 × Thermo protease inhibitor) via incubating on ice for 30 min with vortexing. Cell debris were removed via centrifuging at 12,000 rpm for 20 min. BCA reagent was used to determine protein concentration. 1 mg of cell lysate was incubated with the corresponding primary antibody-conjugated beads at cold room overnight. After incubation, the beads were washed three times with immunoprecipitation buffer, and resuspended in buffer and boiled for 5 min. Proteins were resolved by SDS‒PAGE and analyzed by Western blotting.

### Cycloheximide chase assay

To determine the stability of FBXO45 and Bim, cycloheximide (CHX) chase assay was conducted in breast cancer cells. The transfected breast cancer cells were treated with 100 µg/ml cycloheximide for different time points. Then, cells were harvested and lysed for western blotting to measure protein abundance as describe above.

### Ubiquitination assay

MCF7 and 293T cells were transfected with various plasmid for Flag-Bim, Myc-FBXO45, and His-Ub for 24 h. Then, cells were incubated with 10 µM MG132 for 10 h. Subsequently, cells were harvested and washed three times with PBS. Cells were further lysed in ubiquitination assay buffer and incubated with an anti-Bim antibody overnight at cold room. Immunocomplexes were incubated with Protein A/G plus agarose overnight at cold room. Beads were washed three times with lysis buffer and boiled for 5 min. Then, Western blotting was used to detect ubiquitinated Bim.

### Animal experiments

Six-week-old BALB/c-nu/nu mice were purchased from SLAC Co. Ltd (Shanghai, China) and randomly divided into two groups (5 mice/group) and housed under pathogen-free conditions. MDM MB-231 cells with stable overexpression of FBXO45 were inoculated subcutaneously into the flanks of nude mice and the mammary fat pad, respectively. The mice were inspected to measure the tumor sizes every four days using a digital caliper. The tumor volume was calculated by the standard equation V = A × B^2^ × 0.52 (A: the long diameter; B: the short diameter). The mice were euthanized by compressed carbon dioxide asphyxiation in a chamber at the indicated time points. Tumors were resected and weighted after 40 days of injections. Animal studies were approved by the Institutional Animal Care and Use Committee of Wenzhou Hospital of Integrated Traditional Chinese and Western Medicine. Animal xenograft experiments were performed in accordance with ARRIVE guidelines.

### Statistical analyses

Statistical analyses were performed with GraphPad Prism 8 software. Data were shown as means ± standard deviation. Comparisons between two groups used two-tailed Student’s t test. ANOVA was used for comparisons among multiple groups. Survival was determined by the Kaplan-Meier, and the log-rank test was used for survival comparisons. *P < 0.05, **P < 0.01, and ***P < 0.001 are statistically significant.

## Results

### Downregulation of FBXO45 inhibits proliferation of breast cancer cells

**Fig. 1:**
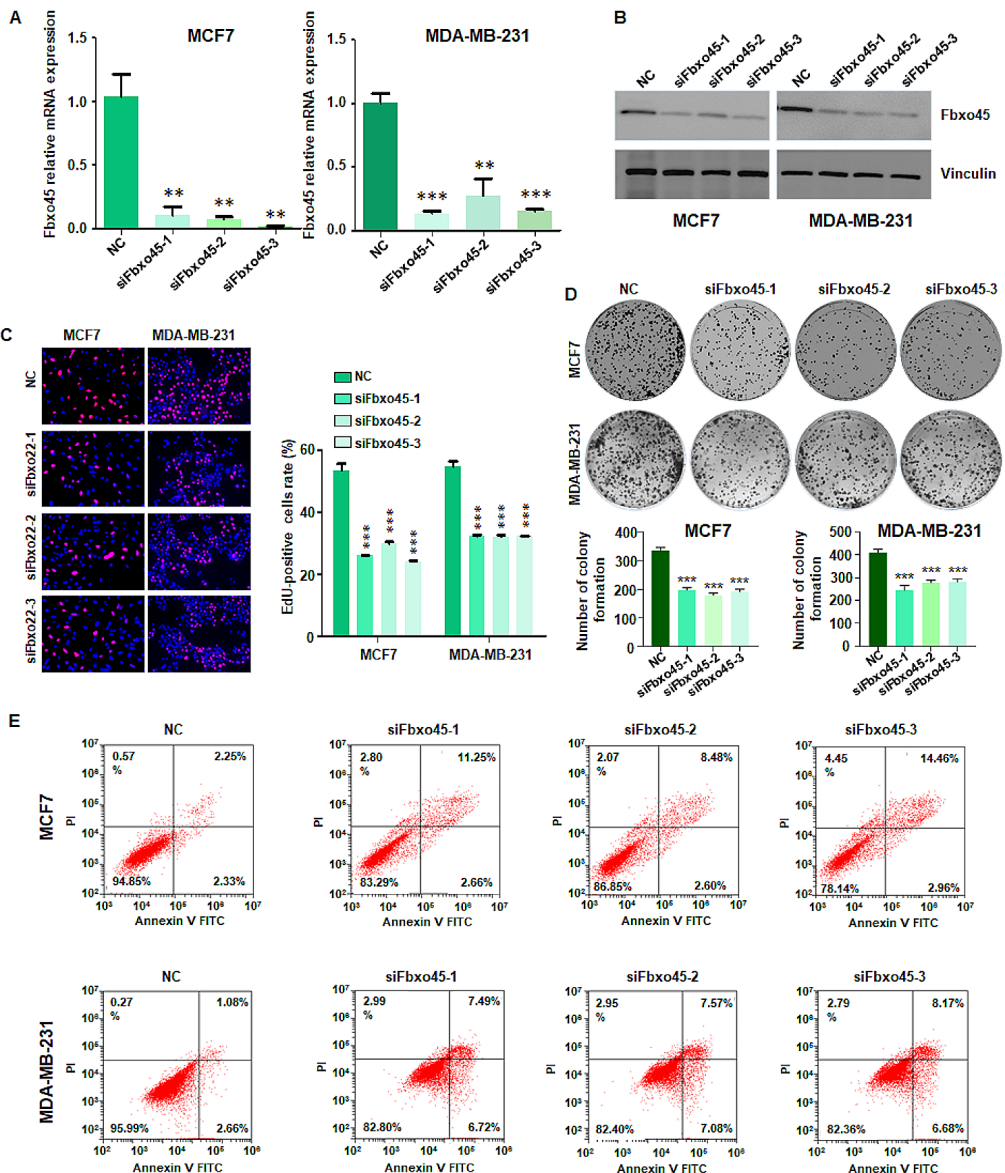
Downregulation of FBXO45 inhibits proliferation in breast cancer cells. (**A**): RT-PCR assay was performed to measure the FBXO45 mRNA levels in siFBXO45-transfected MCF7 and MDA MB-231 cells. **P < 0.01, and ***P < 0.001 vs. control. (**B**): Western blotting assay was performed to test the expression of FBXO45 protein in siFBXO45-transfected MCF7 and MDA MB-231 cells. (**C**): Left panel: EdU assays were performed in breast cancer cells after siFBXO45 transfection. Right panel: Quantitative data were illustrated for left panel. ***P < 0.001 vs. control. (**D**): Colony formation assays were performed to determine the function of siFBXO45 on proliferation of breast cancer cells. (**E**): Cell apoptosis assay was performed in breast cancer cells after siFBXO45 transfection

To test the role of FBXO45 in breast cancer cells, we transfected siFBXO45 into MCF7 and MDA MB-231 cells. Our RT-PCR data showed that siFBXO45 transfection decreased the FBXO45 mRNA levels in MCF7 and MDA MB-231 cells (Fig. 1A). Moreover, our western blotting data further confirmed that siFBXO45 transfection inhibited the expression of FBXO45 protein in MCF7 and MDA MB-231 cells (Fig. 1B and supplementary Fig. 1). Next, we performed the EdU assays in breast cancer cells after siFBXO45 transfection. We found that EdU-positive cells were reduced in MCF7 and MDA MB-231 cells after siFBXO45 transfection (Fig. 1C). This result suggested that inhibition of FBXO45 reduced cell proliferation in breast cancer. Furthermore, we performed colony formation assays to determine the function of FBXO45 on proliferation of breast cancer cells. We found that siFBXO45 transfection reduced colony formation in MCF7 and MDA MB-231 cells (Fig. 1D). It is known that cell proliferation inhibition is often due to promotion of cell apoptotic death in cancer. Hence, we performed cell apoptosis assay in breast cancer cells after FBXO45 modulation. We found that siFBXO45 transfection induced cell apoptosis in MCF7 and MDA MB-231 cells (Fig. 1E). Moreover, we used FBXO45 shRNA transfection and observed that depletion of FBXO45 reduced cell proliferation and colony formation in breast cancer cells (Supplementary Fig. 2A-C). Furthermore, FBXO45 shRNA led to invasion inhibition of breast cancer cells (Supplementary Fig. 2D-E). In addition, we found that FBXO45 depletion reduced the expression of p62 and increased the ratio of LC3 II/LC3 I, indicating that FBXO45 could regulate autophagy (Supplementary Fig. 2F). Together, inhibition of FBXO45 reduced cell proliferation and invasion and induced apoptosis in breast cancer.

### Upregulation of FBXO45 elevates proliferation of breast cancer cells

**Fig. 2:**
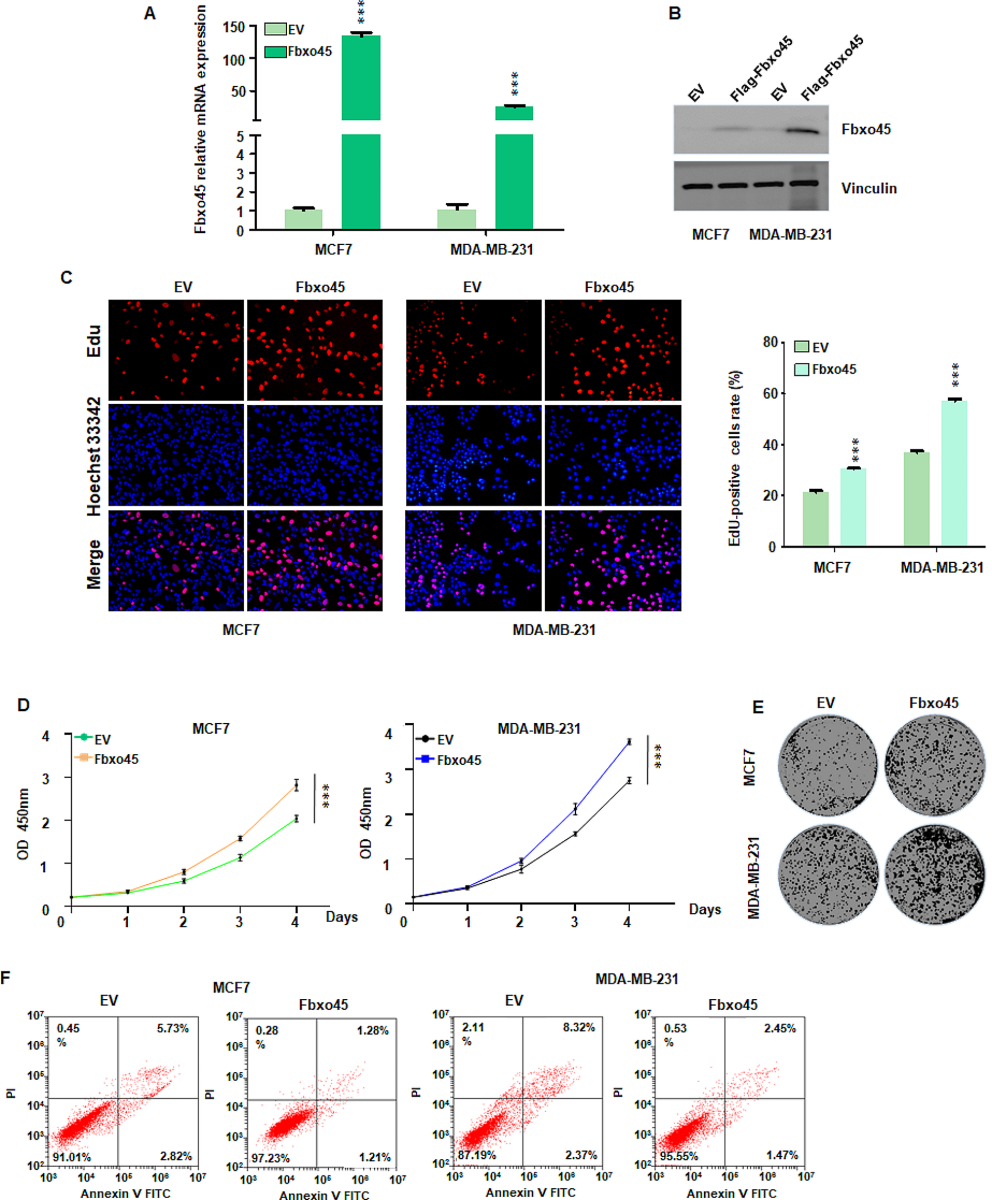
Upregulation of FBXO45 elevates proliferation in breast cancer cells. (**A**): RT-PCR assay was performed to measure the FBXO45 mRNA levels in FBXO45 cDNA-transfected MCF7 and MDA MB-231 cells. ***P < 0.001 vs. control. (**B**): Western blotting assay was performed to test the expression of FBXO45 protein in FBXO45 cDNA-transfected MCF7 and MDA MB-231 cells. (**C**): Left panel: EdU assays were performed in breast cancer cells after FBXO45 cDNA transfection. Right panel: Quantitative data were illustrated for left panel. ***P < 0.001 vs. control. (**D**): CCK-8 assays were performed in breast cancer cells after FBXO45 cDNA transfection. (**E**): Colony formation assays were performed to determine the function of FBXO45 cDNA transfection on proliferation of breast cancer cells. (**F**): Cell apoptosis assay was performed in breast cancer cells after FBXO45 cDNA transfection

Inhibition of FBXO45 reduced proliferation of breast cancer cells and increased cell apoptosis. To confirm this concept, we transfected FBXO45 cDNA into MCF7 and MDA MB-231 cells. Our RT-PCR data showed that FBXO45 cDNA transfection increased the mRNA levels of FBXO45 in both MCF7 and MDA MB-231 cell lines (Fig. 2A). Consistently, our western blotting data showed that FBXO45 cDNA transfection elevated the expression of FBXO45 protein in MCF7 and MDA MB-231 cells (Fig. 2B). Moreover, EdU assay data showed that EdU-positive cells were increased in MCF7 and MDA MB-231 cells after FBXO45 cDNA transfection (Fig. 2C). Furthermore, CCK-8 assay was performed to test whether FBXO45 cDNA transfection regulated cell viability in breast cancer. We found that FBXO45 cDNA transfection increased viability of MCF7 and MDA MB-231 cells (Fig. 2D). Consistently, MCF7 and MDA MB-231 cells with FBXO45 cDNA transfection exhibited promotion of colony formation (Fig. 2E and supplementary Fig. 3A). In addition, FBXO45 cDNA transfection inhibited cell apoptosis in breast cancer cells after FBXO45 cDNA transfection (Fig. 2F and supplementary Fig. 3B). Interestingly, FBXO45 displayed stronger function in colony formation induction and apoptosis inhibition in MCF7 cells than MDA MB-231 cells, which is required for further investigation. Taken together, FBXO45 upregulation promoted cell proliferation and attenuated cell apoptosis in breast cancer.

### FBXO45 interacts with BIM and regulates its expression

**Fig. 3:**
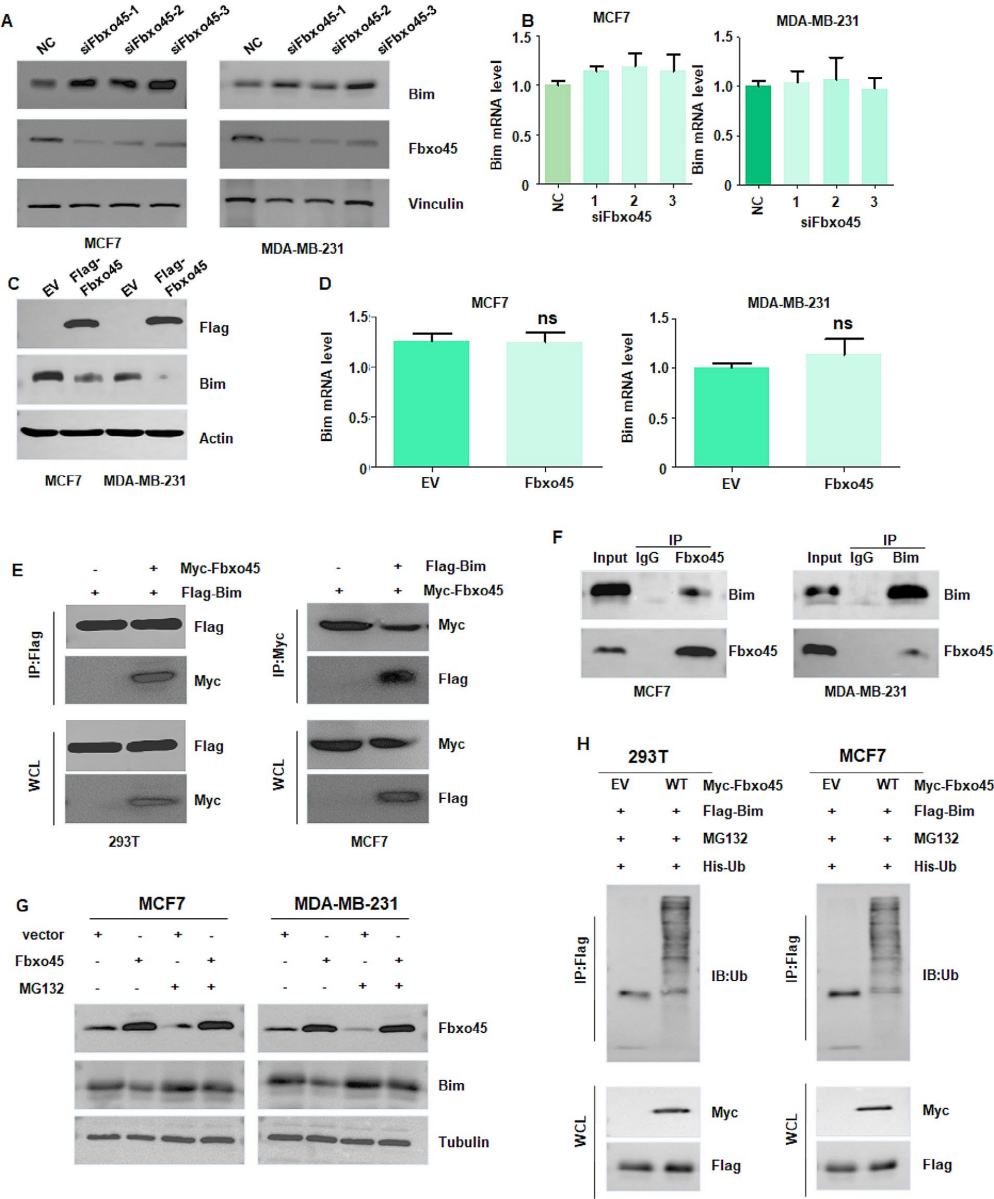
FBXO45 interacts with BIM and regulates its expression. (**A**): Western blotting assay was performed to test the expression of Bim protein in siFBXO45-transfected MCF7 and MDA MB-231 cells. (**B**): RT-PCR assay was performed to measure the Bim mRNA levels in siFBXO45-transfected MCF7 and MDA MB-231 cells. (**C**): Western blotting assay was performed to test the expression of Bim protein in FBXO45 cDNA-transfected MCF7 and MDA MB-231 cells. (**D**): RT-PCR assay was performed to measure the Bim mRNA levels in FBXO45 cDNA-transfected MCF7 and MDA MB-231 cells. (**E**): Exogenous CoIP assay was performed to measure the interaction between FBXO45 and Bim in 293T and MCF7 cells. (**F**): Endogenous IP assay was performed to measure the interaction between FBXO45 and Bim in MCF7 cells and MDA MB-231 cells. (**G**): Western blotting assay was performed to test the expression of Bim protein in FBXO45 cDNA-transfected MCF7 and MDA MB-231 cells after MG132 treatment. (**H**): Ubiquitination assay was performed in breast cancer cells after co-transfection with myc-FBXO45, Flag-Bim and His-Ub

**Fig. 4:**
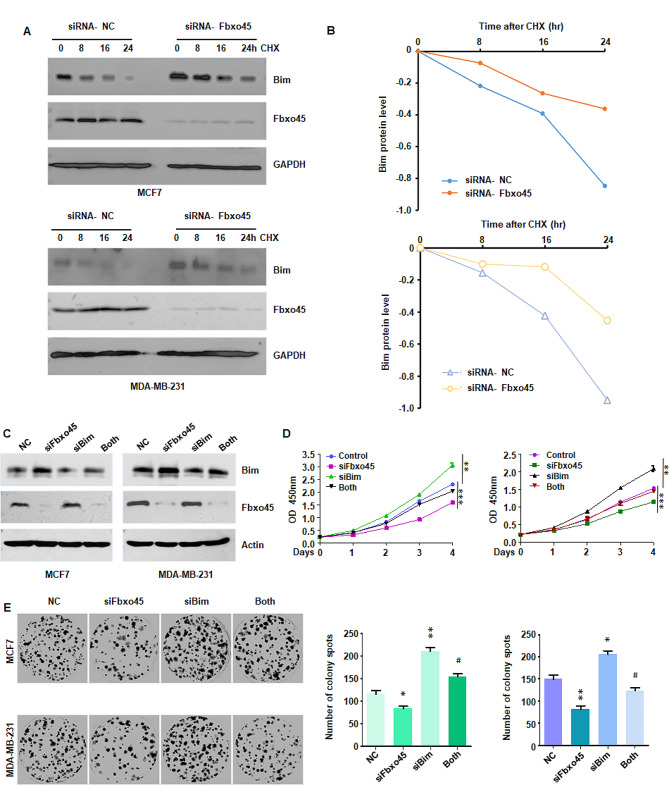
Downregulation of FBXO45 inhibits proliferation via BIM pathway. (**A**): Cycloheximide chase assay was performed to test the half-time of Bim protein in siFBXO45-transfected MCF7 and MDA MB-231 cells. (**B**): Quantitative data were illustrated for panel A. (**C**): Western blotting assay was performed to test the expression of Bim protein in MCF7 and MDA MB-231 cells after co-transfection with siFBXO45 and siBim. (**D**): CCK-8 assay was conducted to measure cell viability in MCF7 and MDA MB-231 cells after co-transfection with siFBXO45 and siBim. (**E**): Left panel: Colony formation assays were performed to determine colony formation ability in MCF7 and MDA MB-231 cells after co-transfection with siFBXO45 and siBim. Right panel: Quantitative data were illustrated for panel E. *P < 0.05, **P < 0.01 vs. control, ^#^*P* < 0.05 vs. siFbxo45 alone or siBim alone

Next, we aimed to determine the molecular mechanism by which FBXO45 promoted cell proliferation and inhibited apoptosis in breast cancer. It is known that BIM plays an essential role in regulation of cell apoptosis in human cancer. Therefore, we measured the expression of BIM at mRNA and protein levels in MCF7 and MDA MB-231 cells after FBXO45 changes. Our western blotting data showed that siFBXO45 transfection increased the expression of BIM protein in MCF7 and MDA MB-231 cells (Fig. 3A and supplementary 4). However, the siFBXO45 transfection failed to change the expression of FBXO45 mRNA levels in MCF7 and MDA MB-231 cells (Fig. 3B). Similarly, FBXO45 cDNA transfection reduced the expression of BIM protein in MCF-7 and MDA MB-231 cells (Fig. 3C), while FBXO45 cDNA transfection did not change the BIM mRNA levels in breast cancer cells (Fig. 3D). Moreover, our IP data showed that FBXO45 interacted with BIM in MCF7, MDA MB-231 and 293T cells (Fig. 3E-F). Furthermore, MG132 treatment abrogated FBXO45-mediated BIM degradation in MCF7 and MDA MB-231 cells (Fig. 3G). Additionally, our ubiquitination assay data showed that FBXO45 cDNA transfection enhanced the ubiquitination of BIM in MCF7 and 293T cells (Fig. 3H). Our cycloheximide chase assay data showed that the half-life of BIM was prolonged after knockdown of endogenous Fbxo45 in MCF7 and MDA MB-231 cells (Fig. 4A-B). Altogether, FBXO45 promoted the ubiquitination and degradation of BIM in breast cancer.

### Downregulation of FBXO45 inhibits proliferation via BIM pathway

We further determined whether downregulation of FBXO45 inhibited proliferation of breast cancer cells via regulation of BIM pathway. MCF7 and MDA MB-231 cells were transfected with siFBXO45, siBIM, or combination. Our western blotting data showed that siBIM transfection inhibited the expression of BIM in breast cancer cells (Fig. 4C). Moreover, siBIM transfection reduced siFBXO45-induced promotion of BIM expression in MCF7 and MDA MB-231 cells (Fig. 4C). CCK-8 assay was performed to measure cell viability in breast cancer cells after siFBXO45 transfection, siBIM transfection, or combination. The results showed that siBIM transfection increased cell viability in MCF7 and MDA MB-231 cells (Fig. 4D). Downregulation of BIM by siRNA transfection abolished siFBXO45-mediated inhibition of viability of MCF7 and MDA MB-231 cells (Fig. 4D). Consistently, siBIM transfection promoted colony formation in MCF7 and MDA MB-231 cells (Fig. 4E). Knockdown of BIM by siRNA rescued siFBXO45-mediated suppression of colony formation in MDA MB-231 and MCF7 cells (Fig. 4E).

### Knockdown of FBXO45 induces apoptosis via BIM pathway

**Fig. 5:**
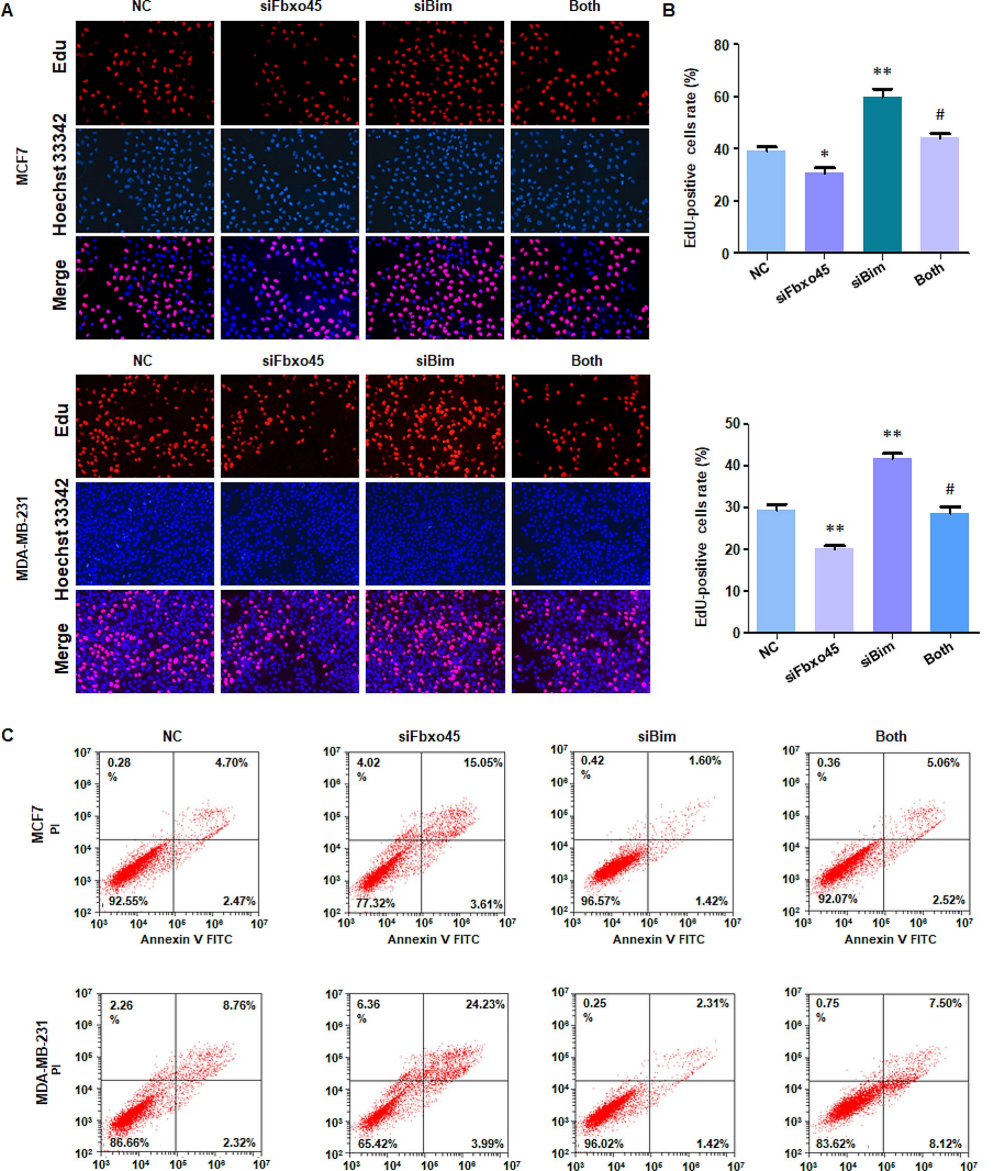
Knockdown of FBXO45 induces apoptosis via BIM pathway. (**A**): EdU assays were performed in breast cancer cells after co-transfection with siFBXO45 and siBim. (**B**): Quantitative data were illustrated for panel A. *P < 0.05, **P < 0.01 vs. control, ^#^*P* < 0.05 vs. siFbxo45 alone or siBim alone. (**C**): Cell apoptosis was measured in MCF7 and MDA MB-231 cells after co-transfection with siFBXO45 and siBim

EdU assay was performed in MCF7 and MDA MB-231 cells transfected with siFBXO45, siBIM, or combination. EdU-positive cell rate was increased in siBIM-treated MCF7 and MDA MB-231 cells (Fig. 5A-B). Moreover, siFBXO45 transfection decreased EdU-positive cell rate in breast cancer cells, which was abrogated by siBIM transfection in MCF7 and MDA MB-231 cells (Fig. 5A-B). BIM is a critical factor in regulation of cell apoptosis in cancer cells. Hence, cell apoptosis was measured in breast cancer cells transfected with siFBXO45, siBIM, or combination. Downregulation of BIM by siRNA in MCF7 and MDA MB-231 inhibited cell apoptosis (Fig. 5C). Further, knockdown of BIM by siRNA rescued siFBXO45-induced cell apoptosis in MCF7 and MDA MB-231 cells (Fig. 5C). taken together, knockdown of FBXO45 induced cell apoptosis via upregulation of BIM in breast cancer.

#### Overexpression of FBXO45 facilitates tumor growth in mice

**Fig. 6:**
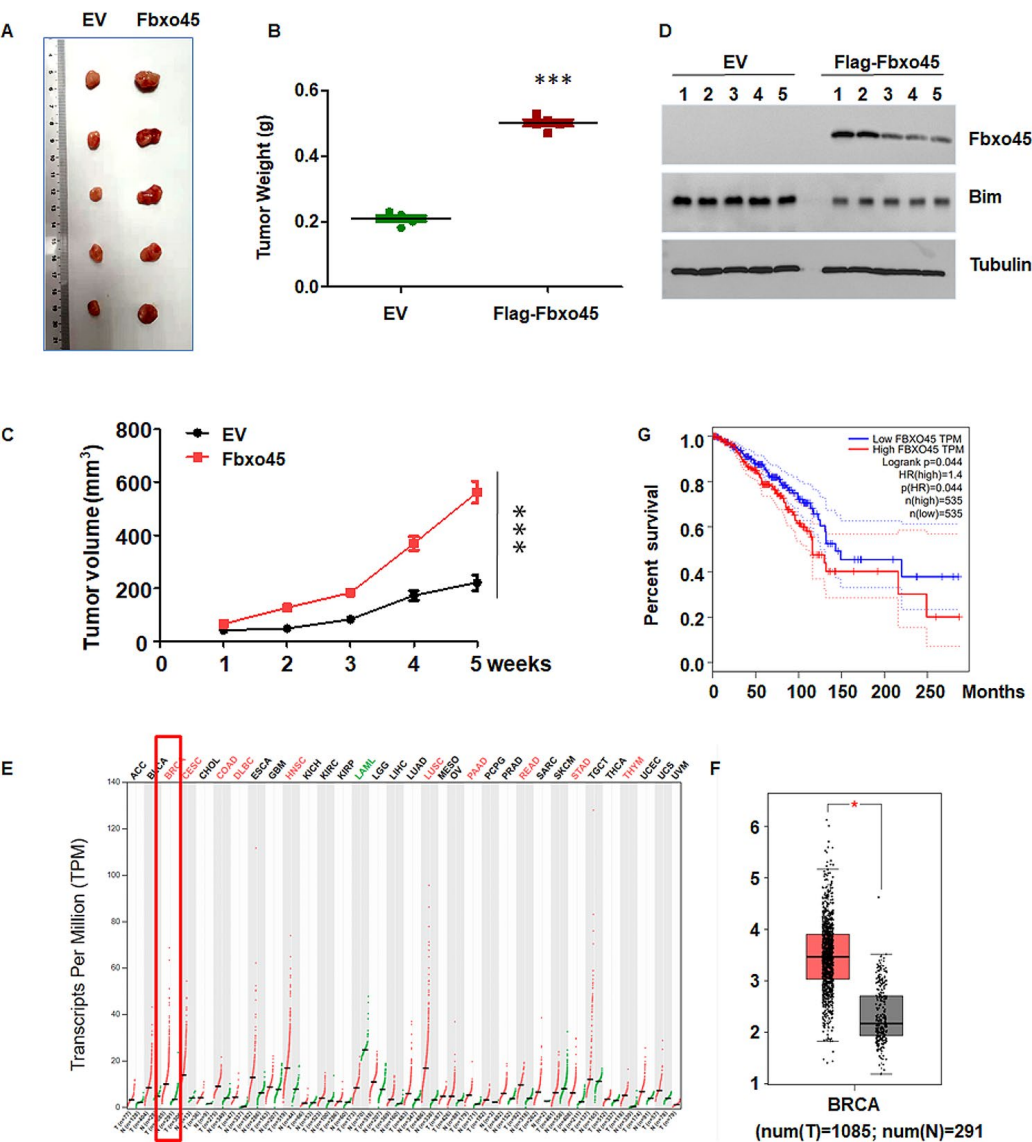
Overexpression of FBXO45 facilitates tumor growth in mice. (**A**): MDM MB-231 cells with stable overexpression of FBXO45 were inoculated subcutaneously into the flanks of nude mice. The tumors were resected and taken a picture. (**B**): The weights of the tumors were illustrated. (**C**): Tumor volumes were illustrated over the time periods. (**D**): Western blotting of the Bim and Fbxo45 protein levels in tumors. (**E**-**F**): GEPIA database showed that FBXO45 was highly expressed in breast cancer patients. (**G**): High expression of FBXO45 were shorter compared with these patients with low expression of FBXO45

To further determine whether FBXO45 regulated tumor growth in mice, MDM MB-231 cells with stable overexpression of FBXO45 were inoculated subcutaneously into the flanks of nude mice. We found that overexpression of FBXO45 in MDA MB-231 cells facilitated tumor growth in breast cancer xenograft mouse model and breast orthotopic tumor model (Fig. 6A-C and supplementary Fig. 5). Tumor mass weights were increased in mice with FBXO45 cDNA transfection group compared with EV group (Fig. 6B). Tumor volumes were larger in FBXO45-overexpressing group compared with control group (Fig. 6C). In mouse tumor tissues, FBXO45-overexpressing group had a lower expression of BIM compared with EV group (Fig. 6D). These data suggested that overexpression of FBXO45 accelerated tumor growth in breast cancer xenograft model.

### FBXO45 expression associates with survival of breast cancer patients

To explore whether FBXO45 expression is correlated with survival of breast cancer patients, we used bioinformatics to analyze the expression of FBXO45 in breast cancer patients. Using GEPIA database, we found that FBXO45 was highly expressed in breast cancer patients compared with normal control (Fig. 6E-F). Moreover, the survival periods of breast cancer patients with high expression of FBXO45 were shorter compared with these patients with low expression of FBXO45 (Fig. 6G). Altogether, FBXO45 expression is correlated with poor survival in breast cancer patients.

## Discussion

Evidence has demonstrated that FBXO45 plays a pivotal role in oncogenesis and progression. FBXO45 expression was elevated and related with shorter overall survival in the squamous-cell lung carcinoma [28]. Wu et al. reported that m6A-induced lncRNA RP11 accelerated the degradation of two mRNAs, FBXO45 and Siah1, which prevented the degradation of ZEB1 and led to dissemination of colorectal cancer [29]. Overexpression of FBXO45 facilitated the ubiquitination of IGF2BP1 and upregulation of PLK1, which conferred to liver tumorigenesis [30]. FBXO45 expression was high and associated with poor prognosis in pancreatic ductal adenocarcinoma (PDAC) [31, 32]. Moreover, bioinformatics analysis showed that FBXO45 could be a potential oncogene in esophageal cancer [33]. FBXO45 was found to be a target of miR-30a-5p in lung squamous cell carcinoma and regulated cell proliferation [34]. FBXO45 mRNA expression was linked with grades, pT stage, pN stage, recurrence, and distant metastasis. FBXO45 was identified as a prognostic biomarker in TMPRSS2-ERG-positive prostate cancer [35].

Wu et al. reported that FBXO45 regulated the ubiquitination and degradation of USP49 and accelerated the tumor progression in pancreatic cancer [36]. FBXO45 maintained ERK activity via targeting NP-STEP46 degradation in lung cancer [37]. Cao et al. reported that lncRNA CACNA1C-AS2 regulated the expression of FBXO45 and PI3K/Akt/mTOR pathways, contributing to suppression of invasion, migration and proliferation in glioma [38]. Wang and colleagues reported that FBXO45 accelerated tumor malignant progression via regulation of ubiquitination and degradation of GGNBP2 in esophageal squamous cell carcinoma [39]. Using a chemically induced mouse model of HCC, one study revealed that FBXO45 promoted fibrosis and inflammation in HCC [40]. RBX1 facilitated tumor metastasis via regulation of FBXO45-TWIST1-dependent degradation in TNBC [41]. DNAJB9 promoted FBXO45-induced degradation of ZEB1 and reduced the tumor metastasis in TNBC [42]. In the current study, we found that FBXO45 promoted cell proliferation and inhibited apoptosis in breast cancer.

Bim, also known as BCL2L11, is a pro-apoptotic protein that belongs to the Bcl-2 protein family [43]. It has been known that Bim plays a crucial role in regulating apoptosis in human cancer cells [44, 45]. Bim has been characterized as a tumor suppressor in tumorigenesis [46]. FoxO3a regulated the expression of Bim and control cell apoptosis in paclitaxel-treated breast cancer cells [47]. Extracellular CD147, a matrix metalloproteinase inducer, inhibited the expression of Bim, leading to Anoikis resistance in breast cancer cells [48]. Bim was critical in phenethyl isothiocyanate-mediated apoptosis in breast cancer cells [49]. One study showed that Bim can be regulated by SNAI2 and inhibit tumor metastasis in breast cancer [50]. Ambra1 (autophagy/Beclin 1 regulator 1) regulated the Akt/FoxO1/Bim pathway and conferred to cell apoptosis and chemosensitivity in breast cancer cells [51, 52]. Uev1A governed the Akt/FoxO1/Bim pathway and enhanced cell survival and chemoresistance in breast cancer cells [53]. Our findings showed that FBXO45 facilitated cell proliferation and inhibited apoptosis via promotion of Bim degradation in breast cancer.

In conclusion, FBXO45 performs oncogenic role in breast cancer via targeting the ubiquitination and degradation of Bim. It is important to clarify several limitations in this study. It is unclear whether FBXO45 enhances tumor growth via regulation of Bim in mice. It is necessary to determine whether FBXO45 expression is negatively associated with Bim expression in breast cancer tissues. Answering these questions will provide the rational for targeting FBXO45 to obtain better outcome in breast cancer treatment. Altogether, targeting FBXO45/Bim axis could be a promising strategy for breast cancer treatment.

### Electronic supplementary material

Below is the link to the electronic supplementary material.

Supplementary Material 1

Supplementary Material 2

## Data Availability

The data of this study are available from the corresponding author upon reasonable request.

## References

[1] Sung H, Ferlay J, Siegel RL, Laversanne M, Soerjomataram I, Jemal A, Bray F (2021). Global Cancer statistics 2020: GLOBOCAN estimates of incidence and Mortality Worldwide for 36 cancers in 185 countries. CA Cancer J Clin.

[2] Siegel RL, Miller KD, Wagle NS, Jemal A (2023). Cancer statistics, 2023. CA Cancer J Clin.

[3] Bianchini G, De Angelis C, Licata L, Gianni L (2022). Treatment landscape of triple-negative breast cancer - expanded options, evolving needs. Nat Rev Clin Oncol.

[4] Swain SM, Shastry M, Hamilton E (2023). Targeting HER2-positive breast cancer: advances and future directions. Nat Rev Drug Discov.

[5] Will M, Liang J, Metcalfe C, Chandarlapaty S (2023). Therapeutic resistance to anti-oestrogen therapy in breast cancer. Nat Rev Cancer.

[6] Pasha N, Turner NC (2021). Understanding and overcoming tumor heterogeneity in metastatic breast cancer treatment. Nat Cancer.

[7] Sirhan Z, Thyagarajan A, Sahu RP (2022). The efficacy of tucatinib-based therapeutic approaches for HER2-positive breast cancer. Mil Med Res.

[8] Li YQ, Sun FZ, Li CX, Mo HN, Zhou YT, Lv D, Zhai JT, Qian HL, Ma F (2023). RARRES2 regulates lipid metabolic reprogramming to mediate the development of brain metastasis in triple negative breast cancer. Mil Med Res.

[9] Harbeck N, Penault-Llorca F, Cortes J, Gnant M, Houssami N, Poortmans P, Ruddy K, Tsang J, Cardoso F (2019). Breast cancer. Nat Rev Dis Primers.

[10] Wang Z, Liu P, Inuzuka H, Wei W (2014). Roles of F-box proteins in cancer. Nat Rev Cancer.

[11] Tekcham DS, Chen D, Liu Y, Ling T, Zhang Y, Chen H, Wang W, Otkur W, Qi H, Xia T (2020). F-box proteins and cancer: an update from functional and regulatory mechanism to therapeutic clinical prospects. Theranostics.

[12] Chen X, Ma J, Wang ZW, Wang Z (2024). The E3 ubiquitin ligases regulate inflammation in cardiovascular diseases. Semin Cell Dev Biol.

[13] Liu J, Chen T, Li S, Liu W, Wang P, Shang G (2022). Targeting matrix metalloproteinases by E3 ubiquitin ligases as a way to regulate the tumor microenvironment for cancer therapy. Semin Cancer Biol.

[14] Yan L, Lin M, Pan S, Assaraf YG, Wang ZW, Zhu X (2020). Emerging roles of F-box proteins in cancer drug resistance. Drug Resist Updat.

[15] Yumimoto K, Yamauchi Y, Nakayama KI. F-Box proteins and Cancer. Cancers (Basel) 2020, 12(5). 10.3390/cancers12051249 PMC7281081 32429232

[16] Li F, Niu M, Qin K, Guo R, Yi Y, Xu J, Li L, Xie S, Fu M, Wen N (2023). FBXL2 promotes E47 protein instability to inhibit breast cancer stemness and paclitaxel resistance. Oncogene.

[17] Xu J, Guo R, Wen N, Li L, Yi Y, Chen J, He Z, Yang J, Xiao ZJ, Niu M (2023). FBXO3 stabilizes USP4 and Twist1 to promote PI3K-mediated breast cancer metastasis. PLoS Biol.

[18] Lin M, Wang ZW, Zhu X (2020). FBXO45 is a potential therapeutic target for cancer therapy. Cell Death Discov.

[19] Peschiaroli A, Scialpi F, Bernassola F, Pagano M, Melino G (2009). The F-box protein FBXO45 promotes the proteasome-dependent degradation of p73. Oncogene.

[20] Chen X, Sahasrabuddhe AA, Szankasi P, Chung F, Basrur V, Rangnekar VM, Pagano M, Lim MS, Elenitoba-Johnson KS (2014). Fbxo45-mediated degradation of the tumor-suppressor Par-4 regulates cancer cell survival. Cell Death Differ.

[21] Wang Z, Wei W (2014). Fbxo45 joins the ‘Par-4’ty in controlling apoptosis of cancer cells. Cell Death Differ.

[22] Hebbar N, Burikhanov R, Shukla N, Qiu S, Zhao Y, Elenitoba-Johnson KSJ, Rangnekar VM (2017). A naturally generated decoy of the prostate apoptosis Response-4 protein overcomes Therapy Resistance in Tumors. Cancer Res.

[23] Richter KT, Kschonsak YT, Vodicska B, Hoffmann I (2020). FBXO45-MYCBP2 regulates mitotic cell fate by targeting FBXW7 for degradation. Cell Death Differ.

[24] Abshire CF, Carroll JL, Dragoi AM (2016). FLASH protects ZEB1 from degradation and supports cancer cells’ epithelial-to-mesenchymal transition. Oncogenesis.

[25] Lin T, Wang D, Chen J, Zhang Z, Zhao Y, Wu Z, Wang Y (2021). IL-24 inhibits the malignancy of human glioblastoma cells via destabilization of Zeb1. Biol Chem.

[26] Kogure N, Yokobori T, Ogata K, Altan B, Mochiki E, Ohno T, Toyomasu Y, Yanai M, Kimura A, Yanoma T (2017). Low expression of FBXO45 is Associated with gastric Cancer progression and poor prognosis. Anticancer Res.

[27] Liu Y, Pan B, Qu W, Cao Y, Li J, Zhao H (2021). Systematic analysis of the expression and prognosis relevance of FBXO family reveals the significance of FBXO1 in human breast cancer. Cancer Cell Int.

[28] Wang K, Qu X, Liu S, Yang X, Bie F, Wang Y, Huang C, Du J (2018). Identification of aberrantly expressed F-box proteins in squamous-cell lung carcinoma. J Cancer Res Clin Oncol.

[29] Wu Y, Yang X, Chen Z, Tian L, Jiang G, Chen F, Li J, An P, Lu L, Luo N (2019). M(6)A-induced lncRNA RP11 triggers the dissemination of colorectal cancer cells via upregulation of Zeb1. Mol Cancer.

[30] Lin XT, Yu HQ, Fang L, Tan Y, Liu ZY, Wu D, Zhang J, Xiong HJ, Xie CM. Elevated FBXO45 promotes liver tumorigenesis through enhancing IGF2BP1 ubiquitination and subsequent PLK1 upregulation. Elife 2021, 10. 10.7554/eLife.70715 PMC8641947 34779401

[31] Shang X, Shi LE, Taule D, Zhu ZZ (2021). A Novel miRNA-mRNA Axis involves in regulating Transcriptional disorders in pancreatic adenocarcinoma. Cancer Manag Res.

[32] Zhang Y, Liu Q, Cui M, Wang M, Hua S, Gao J, Liao Q (2021). Comprehensive Analysis of expression, Prognostic Value, and Immune Infiltration for Ubiquitination-related FBXOs in pancreatic ductal adenocarcinoma. Front Immunol.

[33] Zhang J, Zhou Y, Zhang B, Wang C, Chen B, Ma H (2021). Bioinformatics analysis identifying FBXO45 gene as a potential oncogene in esophageal cancer. J Gastrointest Oncol.

[34] Zeng F, You S, Dai X (2022). MiR-30a-5p hampers proliferation of lung squamous cell carcinoma through targeting FBXO45. Histol Histopathol.

[35] von Danwitz M, Klumper N, Bernhardt M, Cox A, Krausewitz P, Alajati A, Kristiansen G, Ritter M, Ellinger J, Stein J. Identification of F-Box/SPRY domain-containing protein 1 (FBXO45) as a prognostic biomarker for TMPRSS2-ERG-Positive primary prostate cancers. Cancers (Basel) 2023, 15(6). 10.3390/cancers15061890 PMC10046786 36980776

[36] Wu L, Yu K, Chen K, Zhu X, Yang Z, Wang Q, Gao J, Wang Y, Cao T, Xu H (2022). Fbxo45 facilitates pancreatic carcinoma progression by targeting USP49 for ubiquitination and degradation. Cell Death Dis.

[37] Wang Q, Xu C, Cai R, An W, Yuan H, Xu M (2022). Fbxo45-mediated NP-STEP(46) degradation via K6-linked ubiquitination sustains ERK activity in lung cancer. Mol Oncol.

[38] Cao T, Cui Y, Wang Y, Wu L, Yu K, Chen K, Xia J, Li Y, Wang ZP, Ma J (2022). CACNA1C-AS2 inhibits cell proliferation and suppresses cell migration and invasion via targeting FBXO45 and PI3K/AKT/mTOR pathways in glioma. Apoptosis.

[39] Wang Q, Wu L, Cao R, Gao J, Chai D, Qin Y, Ma L, Wu S, Tao Y, Ma J (2022). Fbxo45 promotes the malignant development of esophageal squamous cell carcinoma by targeting GGNBP2 for ubiquitination and degradation. Oncogene.

[40] Zhang J, Lin XT, Fang L, Xie CM (2023). In vivo analysis of FBXO45-mediated fibrosis and liver tumorigenesis in a chemically induced mouse model of hepatocellular carcinoma. STAR Protoc.

[41] Shao J, Feng Q, Jiang W, Yang Y, Liu Z, Li L, Yang W, Zou Y (2022). E3 ubiquitin ligase RBX1 drives the metastasis of triple negative breast cancer through a FBXO45-TWIST1-dependent degradation mechanism. Aging.

[42] Kim HY, Kim YM, Hong S (2021). DNAJB9 suppresses the metastasis of triple-negative breast cancer by promoting FBXO45-mediated degradation of ZEB1. Cell Death Dis.

[43] Shukla S, Saxena S, Singh BK, Kakkar P (2017). BH3-only protein BIM: an emerging target in chemotherapy. Eur J Cell Biol.

[44] Akiyama T, Dass CR, Choong PF (2009). Bim-targeted cancer therapy: a link between drug action and underlying molecular changes. Mol Cancer Ther.

[45] Faber AC, Ebi H, Costa C, Engelman JA (2012). Apoptosis in targeted therapy responses: the role of BIM. Adv Pharmacol.

[46] Sionov RV, Vlahopoulos SA, Granot Z (2015). Regulation of Bim in Health and Disease. Oncotarget.

[47] Sunters A, Fernandez de Mattos S, Stahl M, Brosens JJ, Zoumpoulidou G, Saunders CA, Coffer PJ, Medema RH, Coombes RC, Lam EW (2003). FoxO3a transcriptional regulation of Bim controls apoptosis in paclitaxel-treated breast cancer cell lines. J Biol Chem.

[48] Yang JM, O’Neill P, Jin W, Foty R, Medina DJ, Xu Z, Lomas M, Arndt GM, Tang Y, Nakada M (2006). Extracellular matrix metalloproteinase inducer (CD147) confers resistance of breast cancer cells to anoikis through inhibition of Bim. J Biol Chem.

[49] Hahm ER, Singh SV (2012). Bim contributes to phenethyl isothiocyanate-induced apoptosis in breast cancer cells. Mol Carcinog.

[50] Merino D, Best SA, Asselin-Labat ML, Vaillant F, Pal B, Dickins RA, Anderson RL, Strasser A, Bouillet P, Lindeman GJ (2015). Pro-apoptotic bim suppresses breast tumor cell metastasis and is a target gene of SNAI2. Oncogene.

[51] Sun WL, He LY, Liang L, Liu SY, Luo J, Lv ML, Cai ZW (2022). Ambra1 regulates apoptosis and chemosensitivity in breast cancer cells through the Akt-FoxO1-Bim pathway. Apoptosis.

[52] Sun WL, Wang L, Luo J, Zhu HW, Cai ZW (2019). Ambra1 inhibits paclitaxel-induced apoptosis in breast cancer cells by modulating the Bim/mitochondrial pathway. Neoplasma.

[53] Wu Z, Niu T, Xiao W (2019). Uev1A promotes breast cancer cell survival and chemoresistance through the AKT-FOXO1-BIM pathway. Cancer Cell Int.

