# Supplementary material for: Fbxo45 facilitates the malignant progression of breast cancer by targeting Bim for ubiquitination and degradation
Source: BMC Cancer. 2024 May 21;24:619. doi: 10.1186/s12885-024-12382-8 (PMC11110447; doi:10.1186/s12885-024-12382-8)
Supplement: Supplementary file 1 — Supplementary Material 1 [file 12885_2024_12382_MOESM1_ESM.pdf]

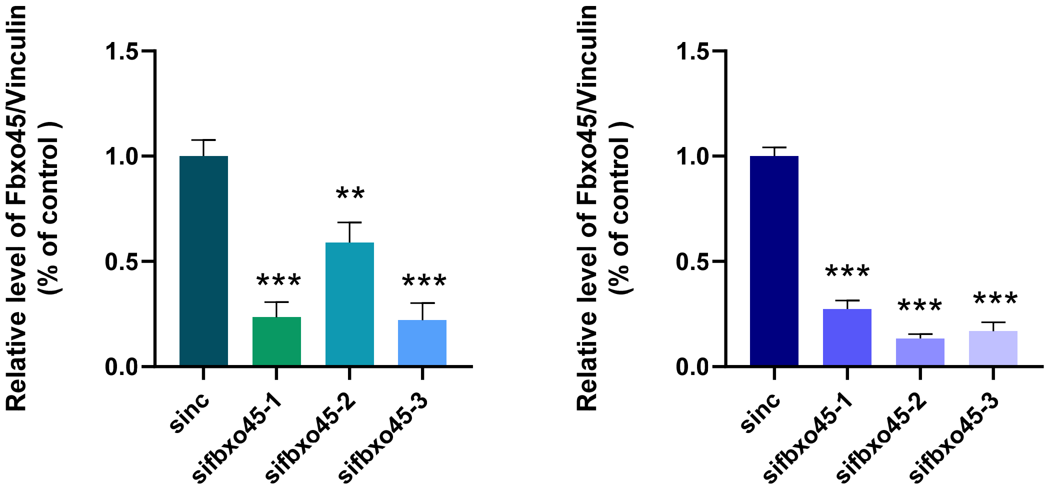

Supplementary figure 1: Quantitative data were illustrated for Figure 1B. **P < 0.05 vs control; ***P < 0.001 vs control.

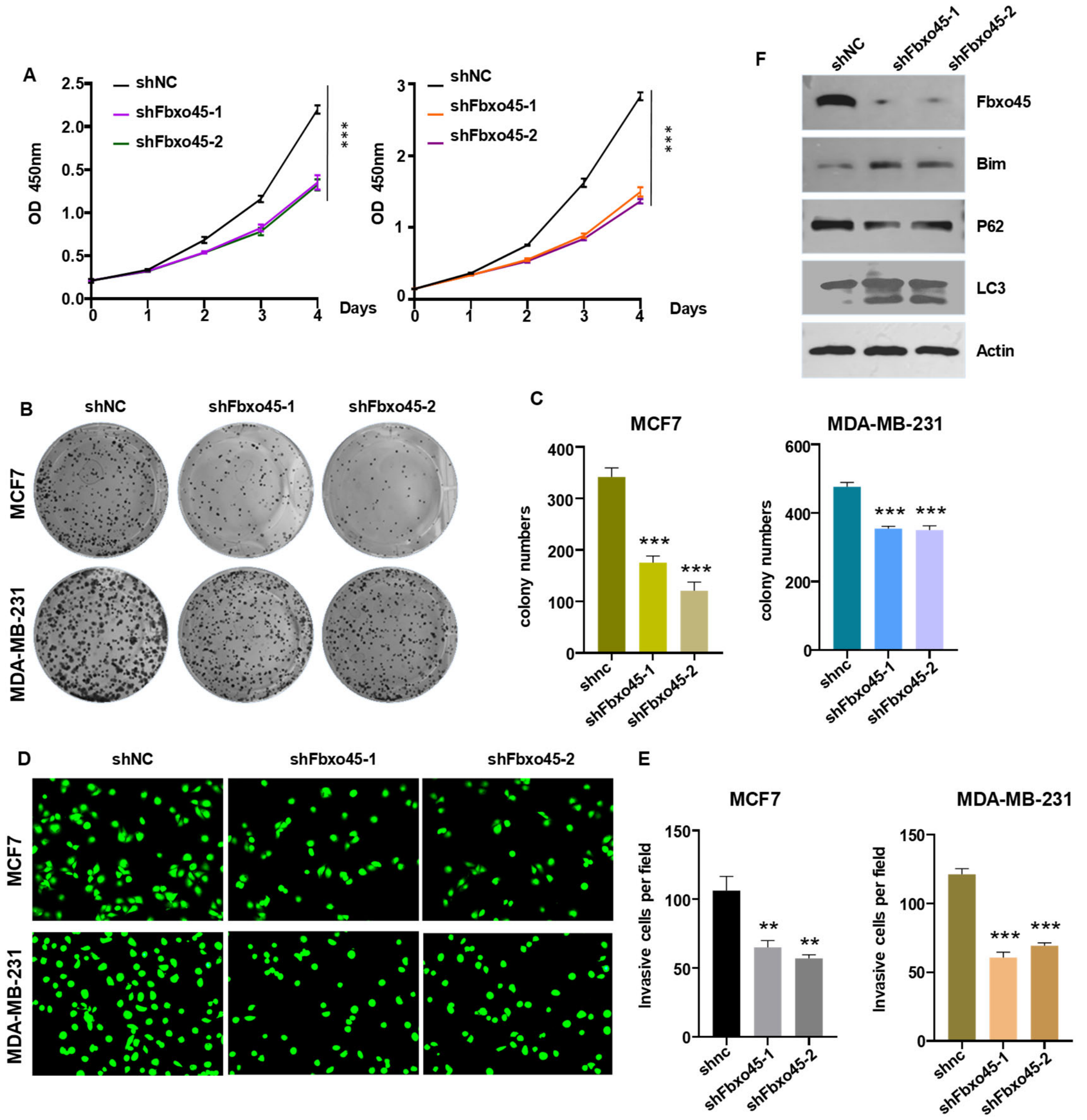

Supplementary figure 2: Depletion of FBXO45 inhibited cell proliferation and invasion.
A, CCK8 assay was performed in breast cancer cells after shFBXO45 transfection.
B: Colony formation assays were performed to determine the function of shFBXO45 on proliferation of breast cancer cells.
C: Quantitative data were illustrated for left panel.
D. Cell invasion assay was performed in breast cancer cells after shFBXO45 transfection. **P < 0.05 vs control; ***P < 0.001 vs control.
E: Western blotting assay was performed in breast cancer cells after shFBXO45 transfection.

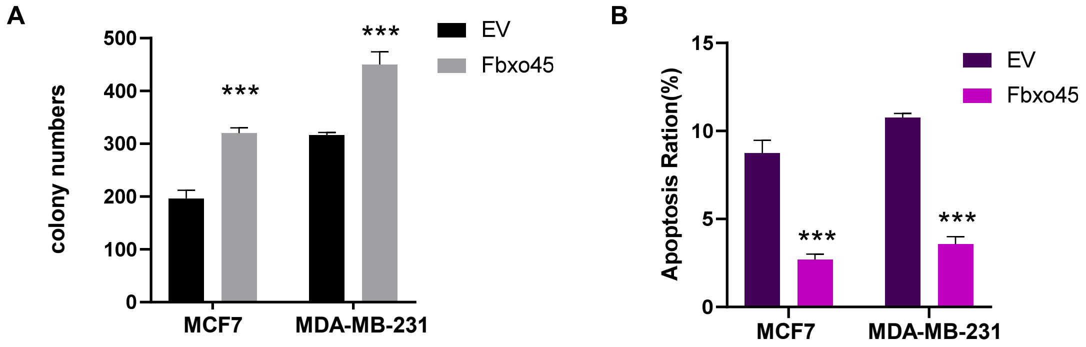

Supplementary figure 3: FBXO45 overexpression increased colony formation and inhibited apoptosis. A, Quantitative data were illustrated for Figure 2E. B, Quantitative data were illustrated for Figure 2F. ***P < 0.001 vs control.

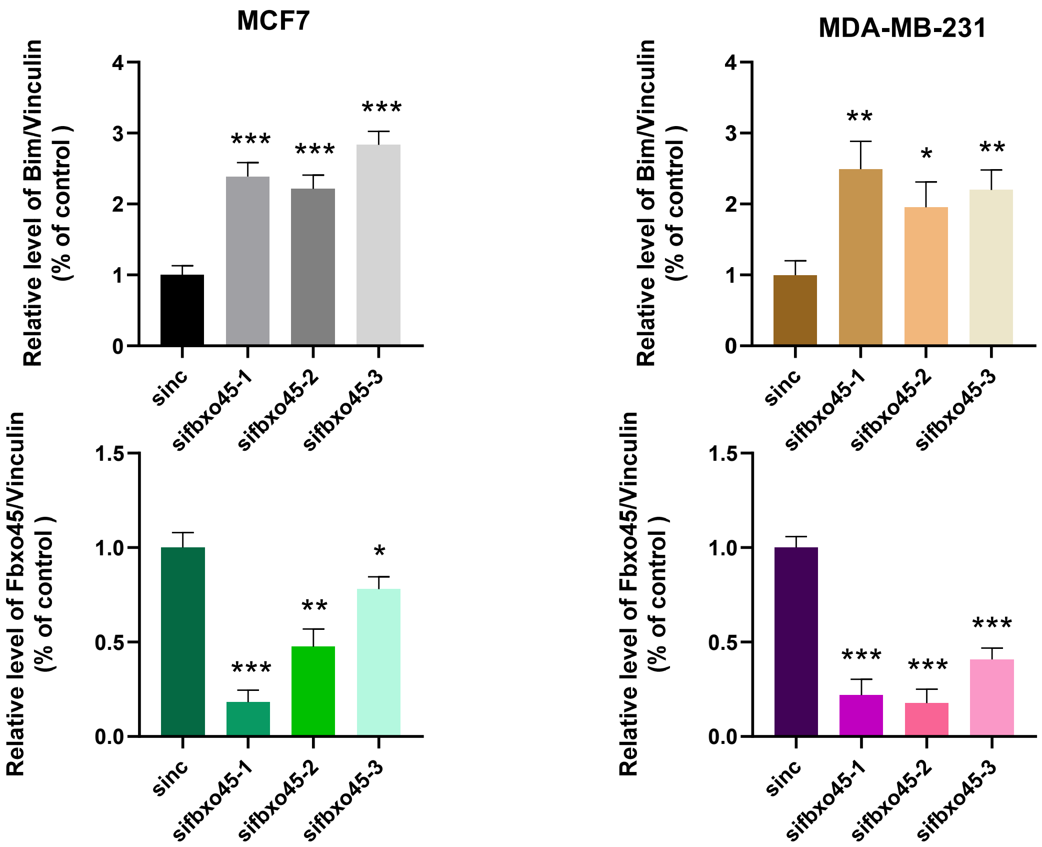

Supplementary figure 4: Quantitative data were illustrated for Figure 3A. **P < 0.05 vs control; ***P < 0.001 vs control.

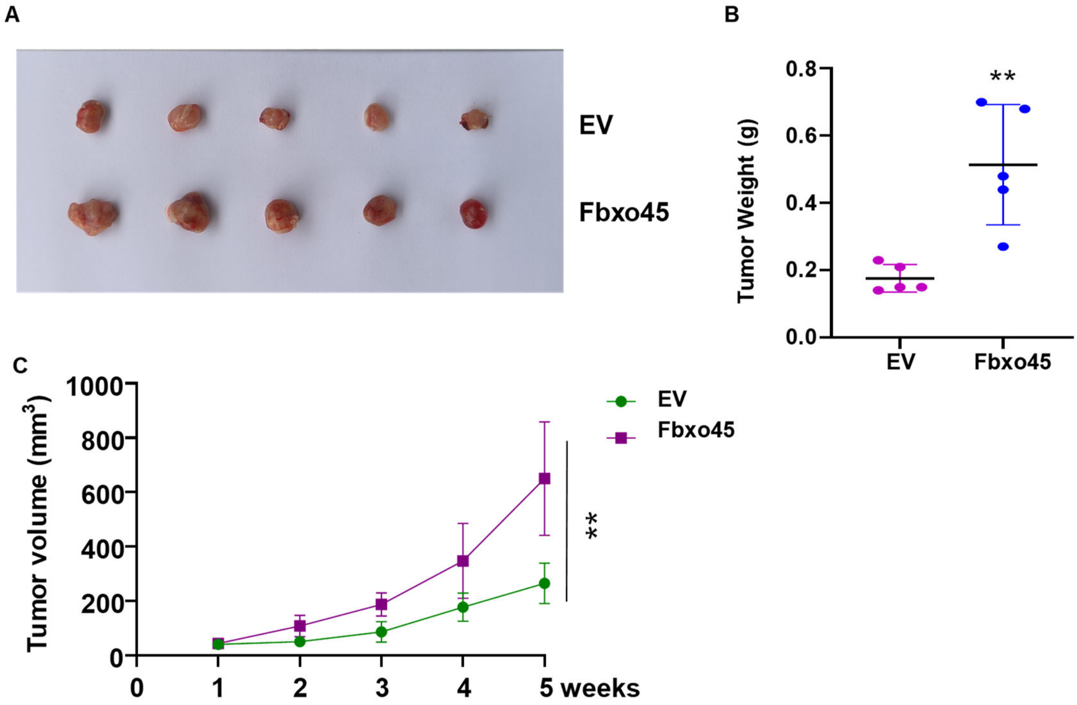

Supplementary figure 5: Overexpression of FBXO45 facilitates tumor growth in mice.

A: MDM MB-231 cells with stable overexpression of FBXO45 were inoculated into the mammary fat pad of nude mice. The tumors were resected and taken a picture.

B: The weights of the tumors were illustrated.

C: Tumor volumes were illustrated over the time periods.
