# Supplementary material for: Fbxo45 facilitates the malignant progression of breast cancer by targeting Bim for ubiquitination and degradation
Source: BMC Cancer. 2024 May 21;24:619. doi: 10.1186/s12885-024-12382-8 (PMC11110447; doi:10.1186/s12885-024-12382-8)
Supplement: Supplementary file 2 — Supplementary Material 2 [file 12885_2024_12382_MOESM2_ESM.pdf]

Fig 1B

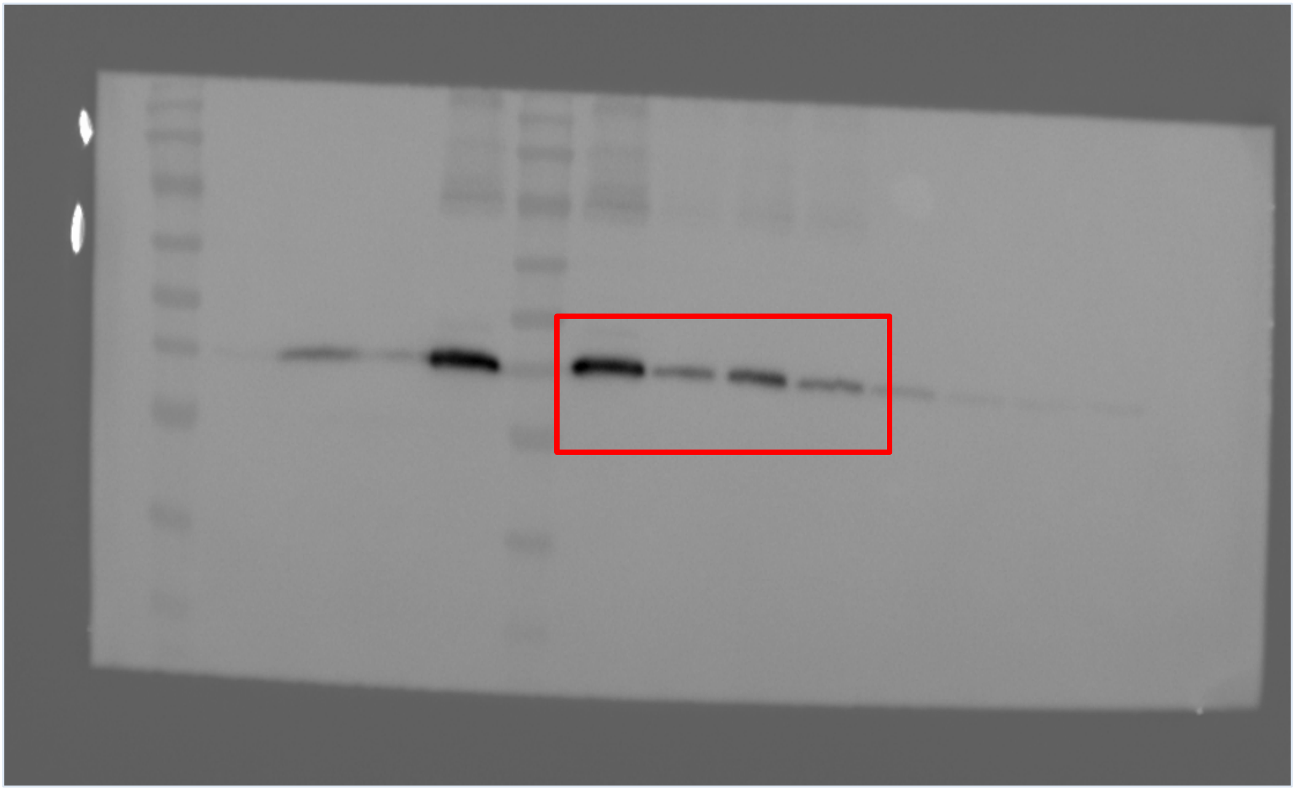

Fbxo45

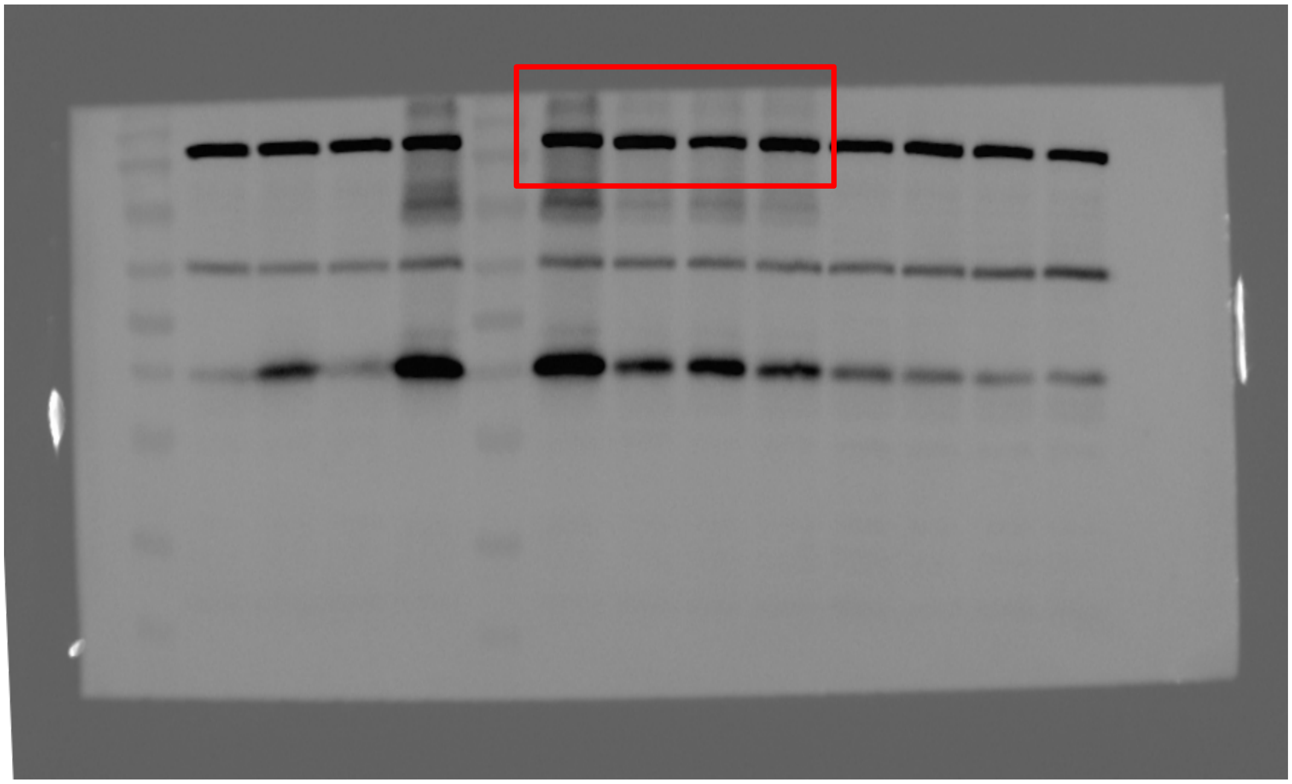

Vinculin

Fig 2B

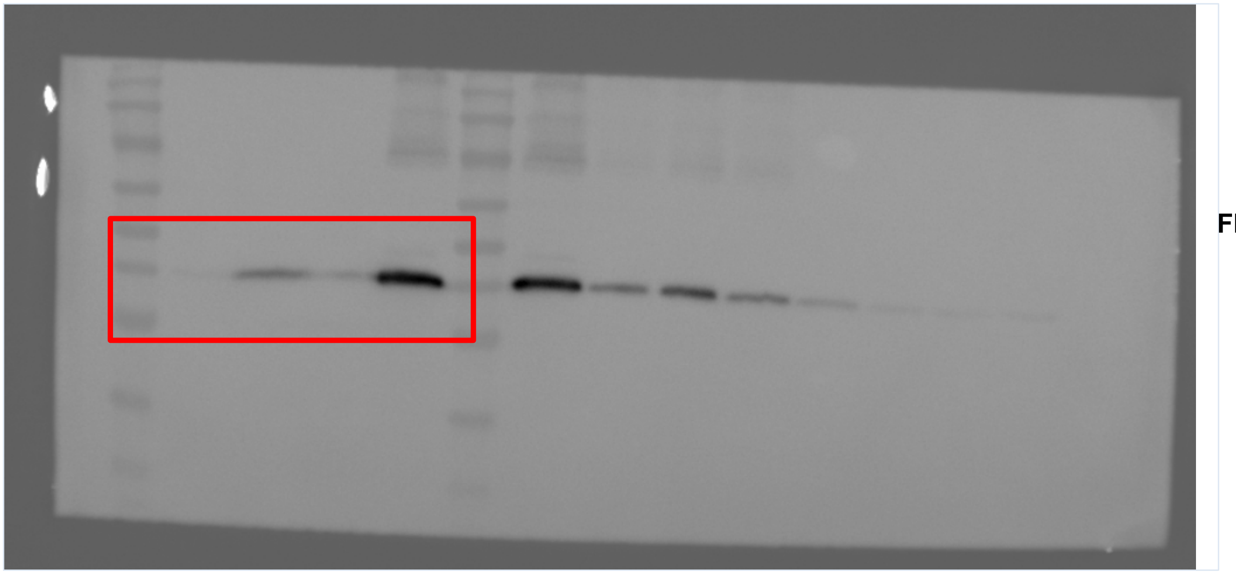

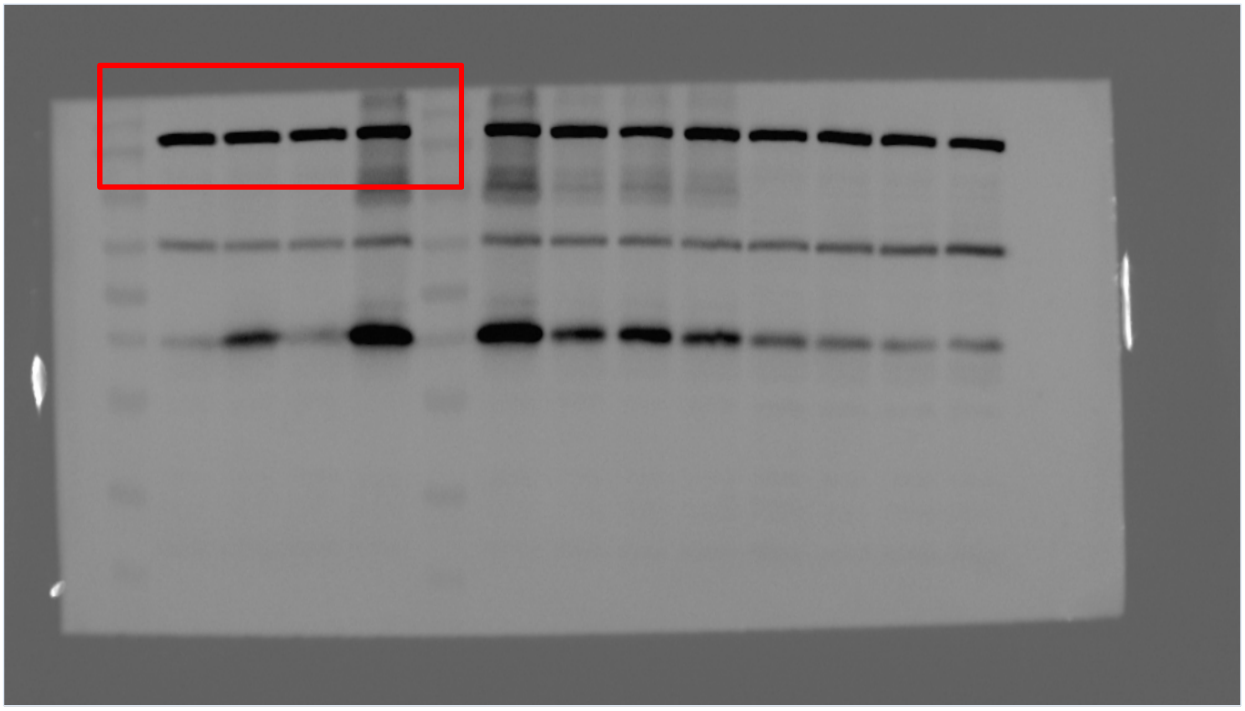

Fig 3A

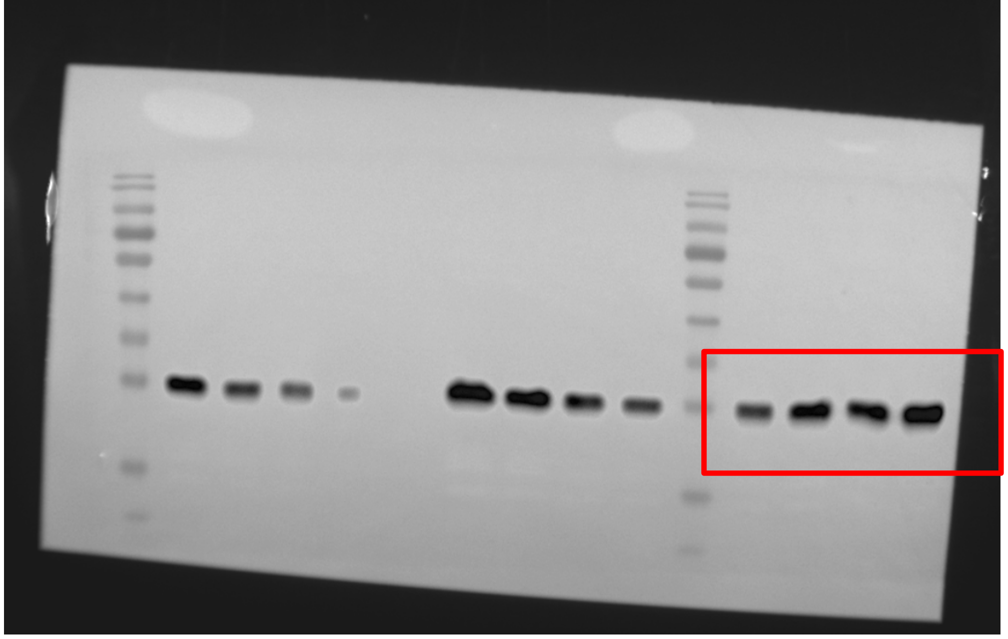

Bim

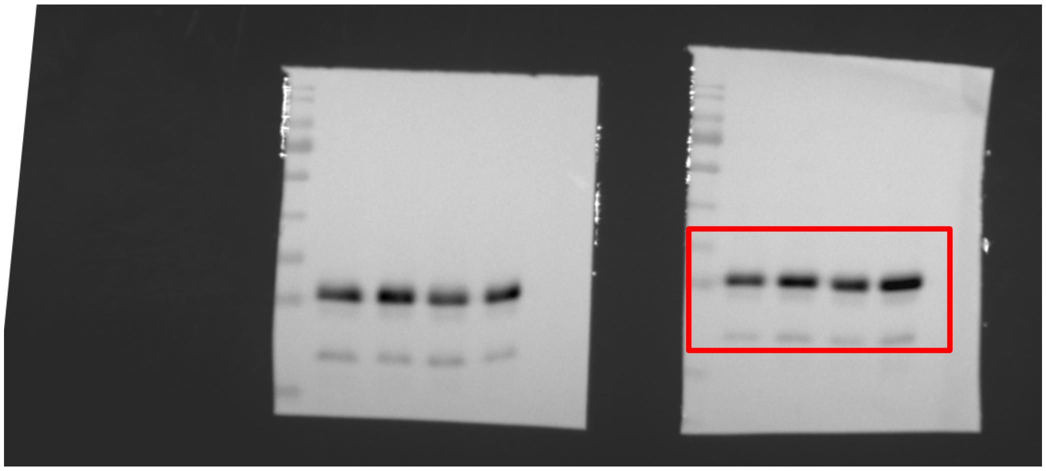

Bim

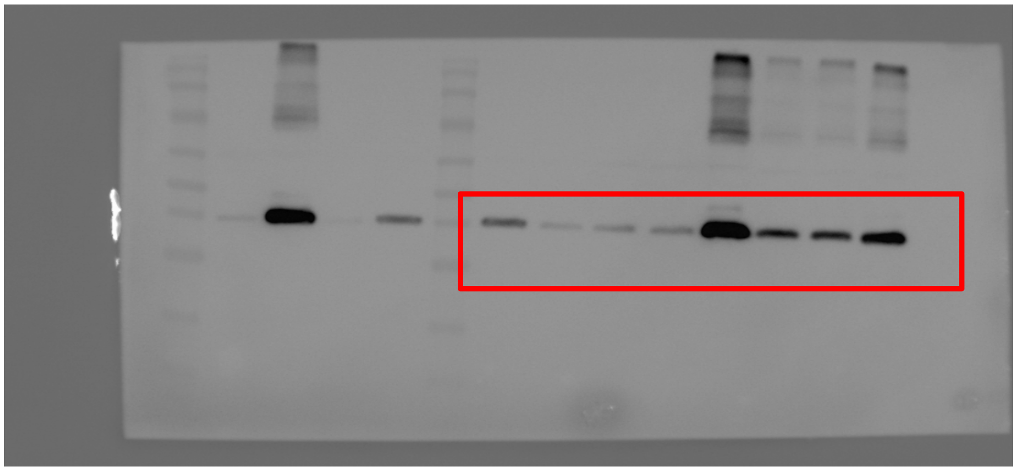

Fbxo45

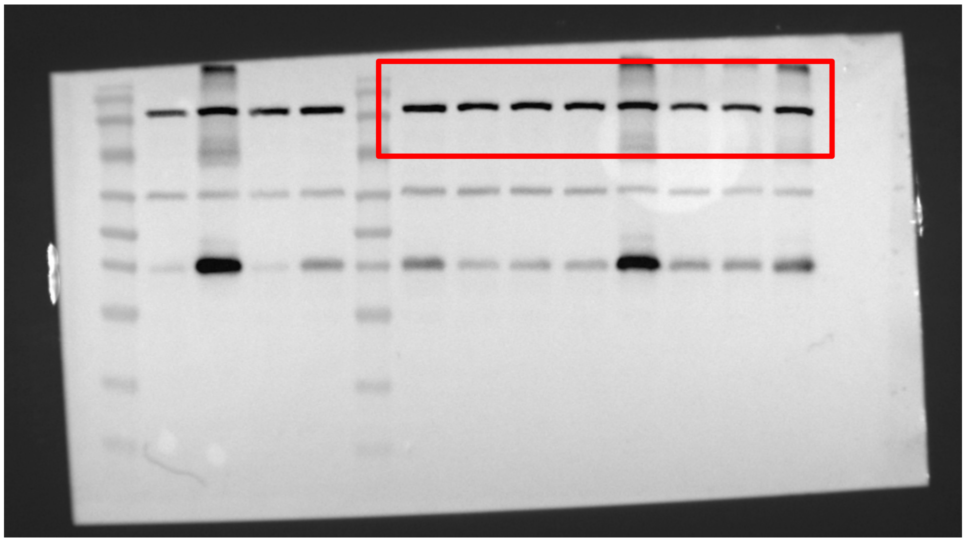

Vinculin

Fig 3C

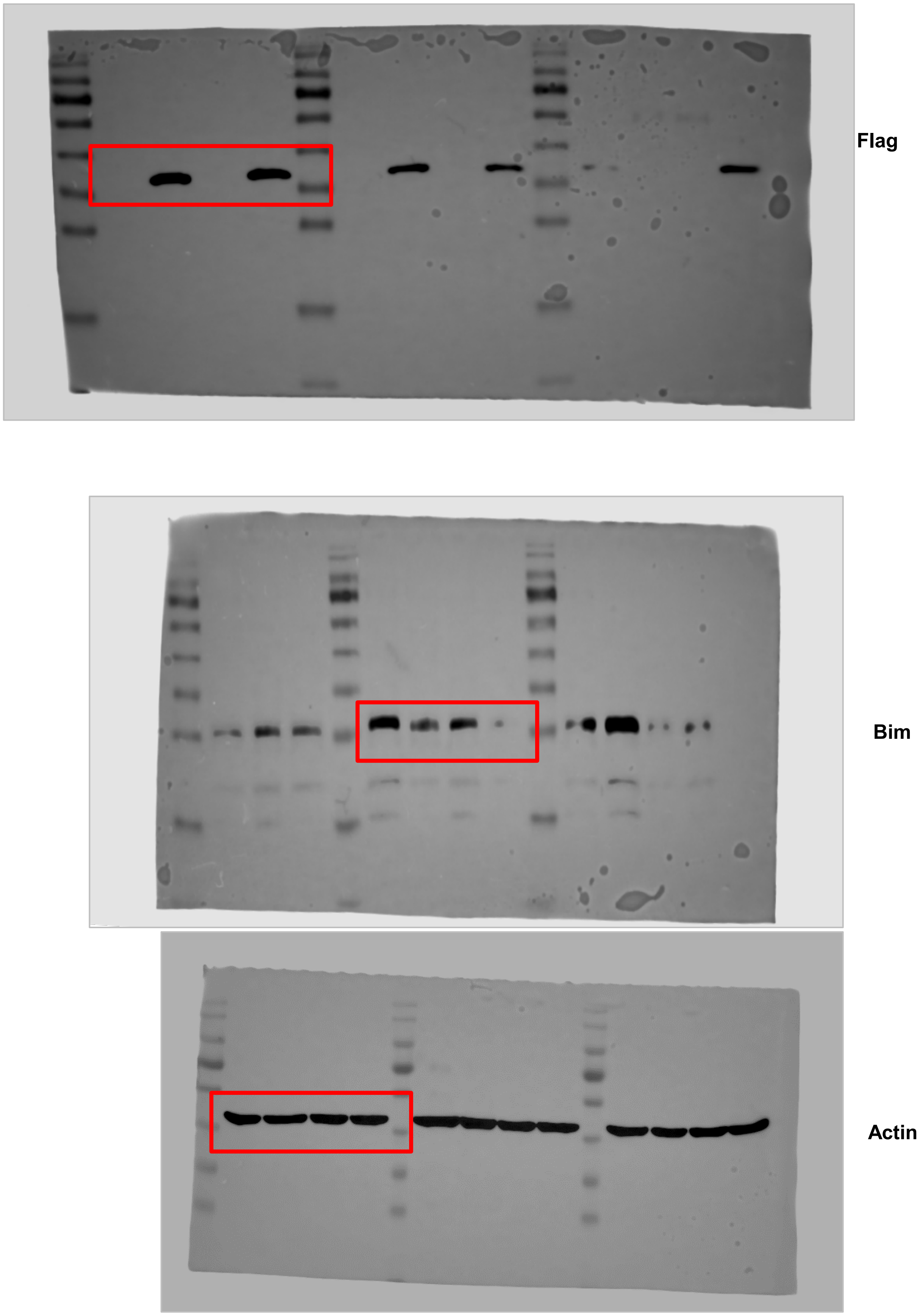

Fig 3E

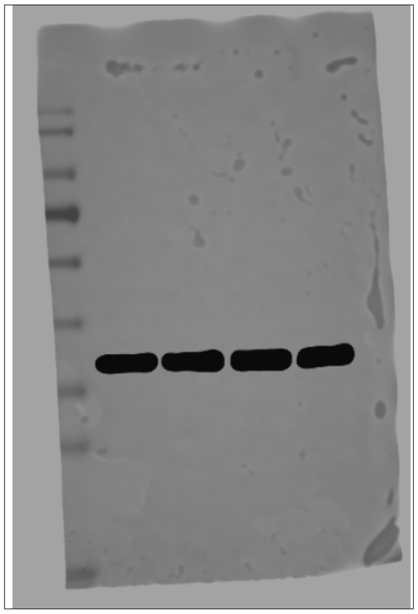

Flag

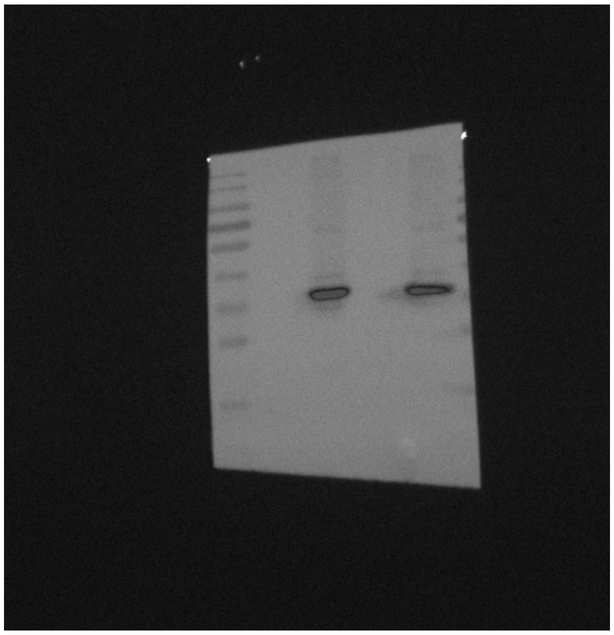

Myc

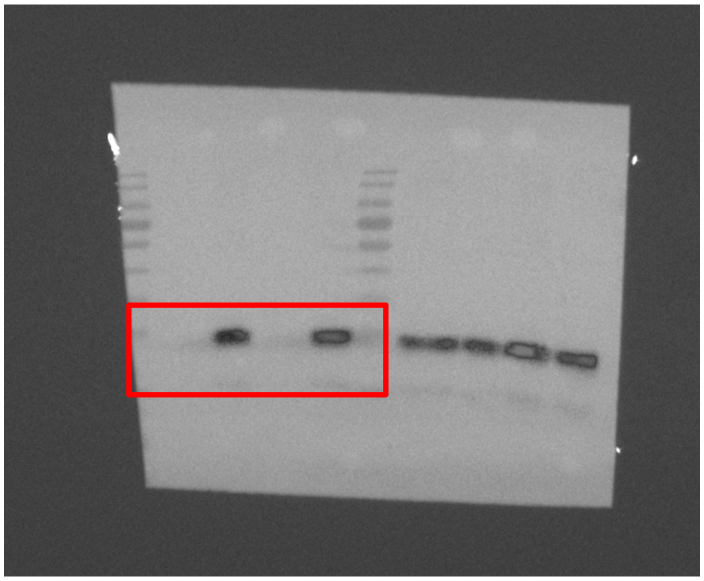

Flag

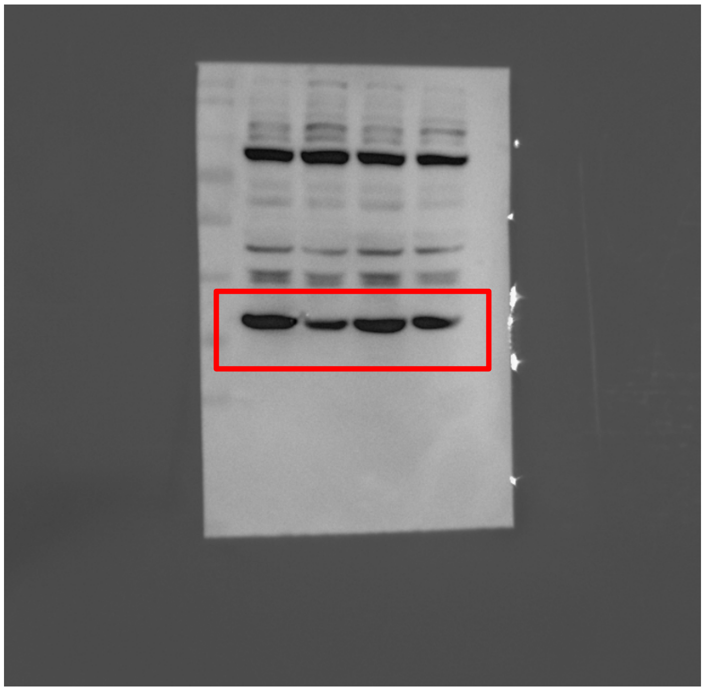

Myc

Fig 3F

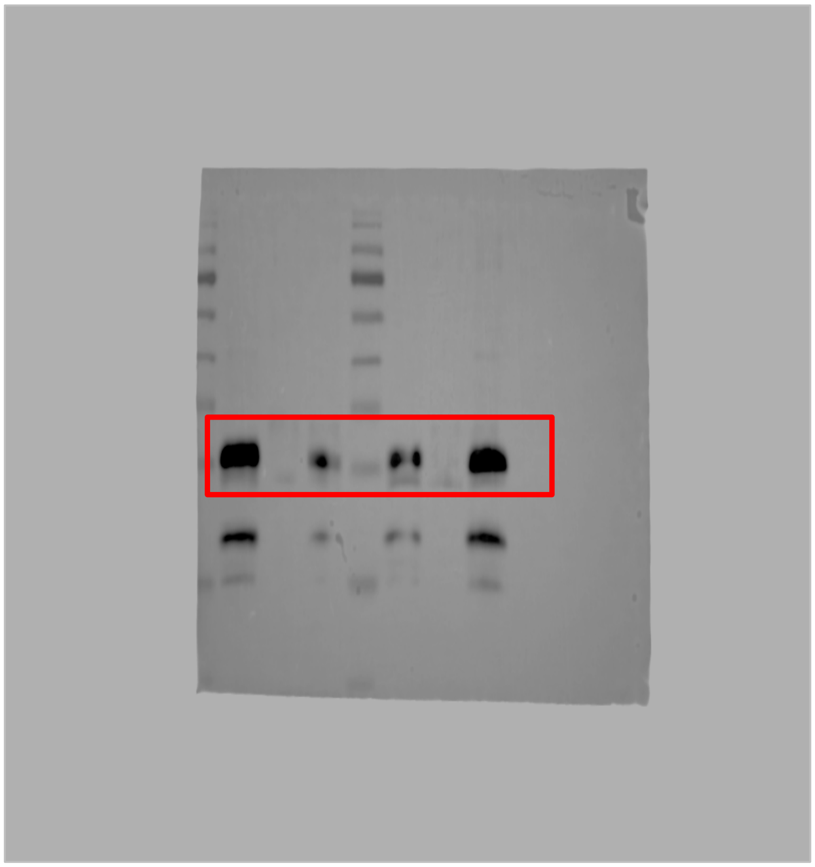

Bim

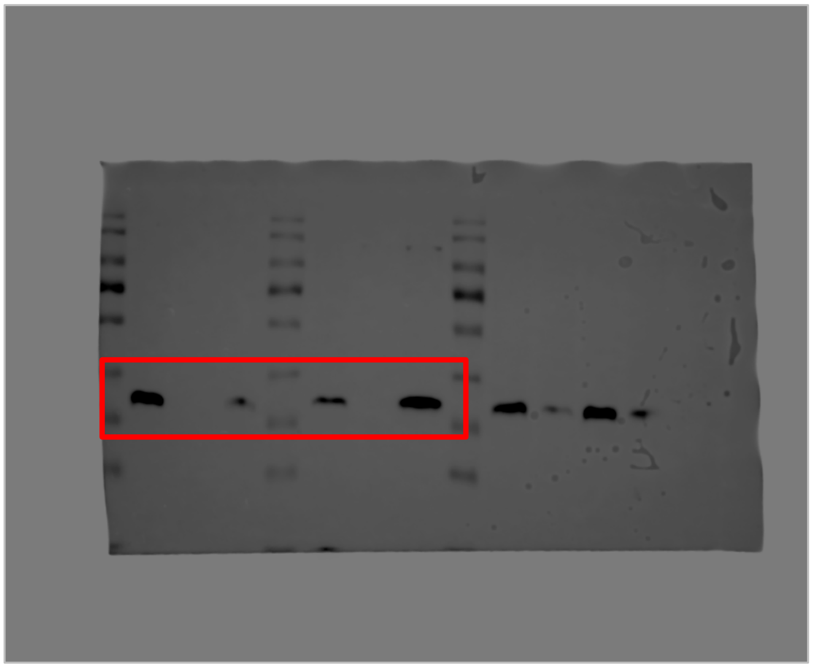

Fbxo45

Fig 3G

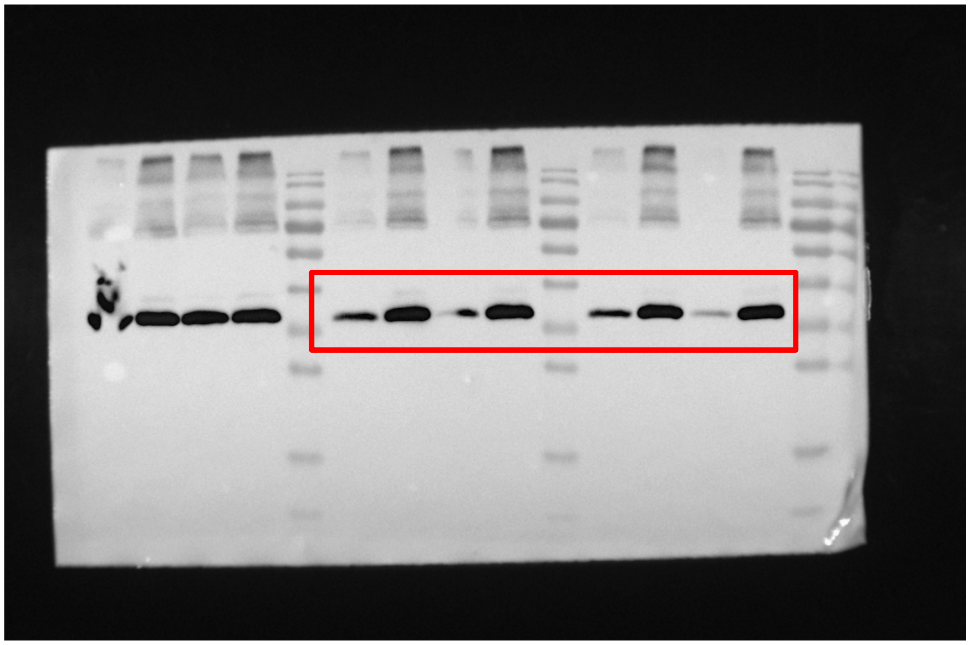

Fbxo45

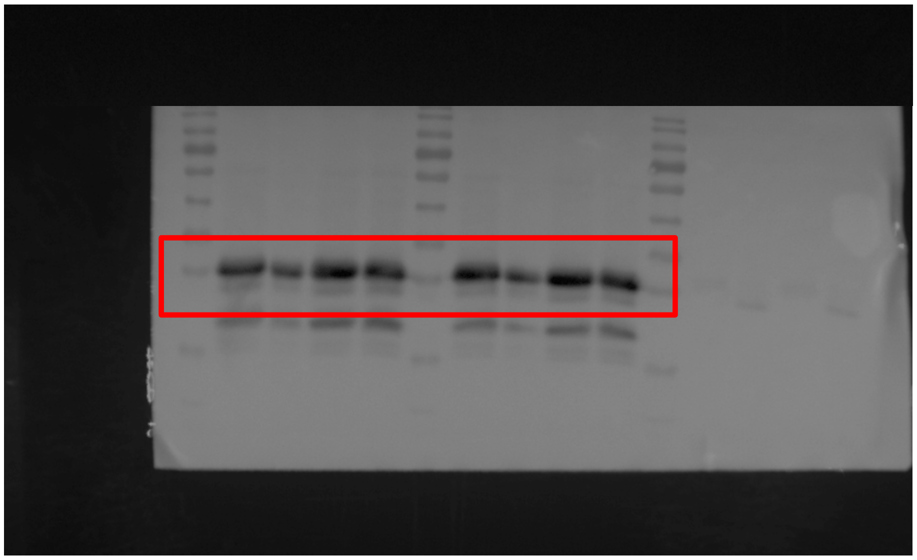

Bim

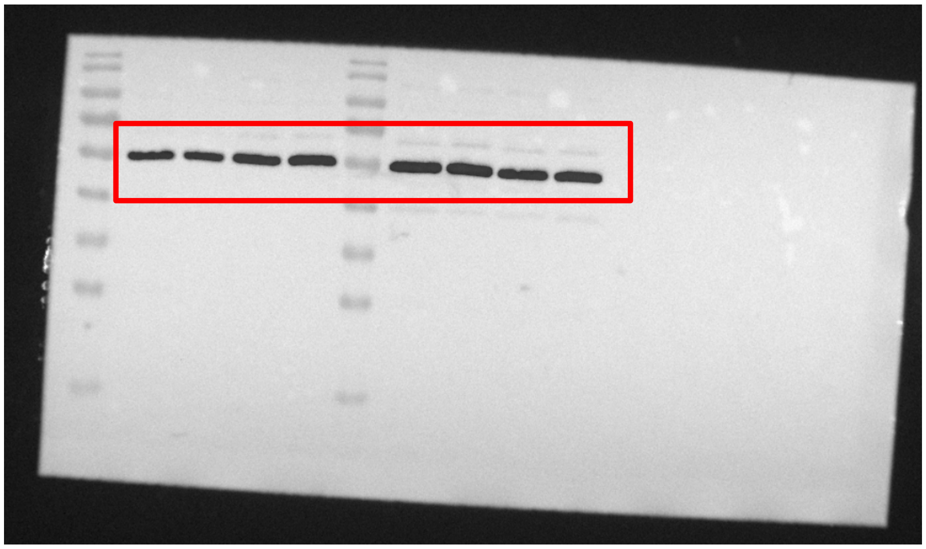

Tubulin

Fig 3H

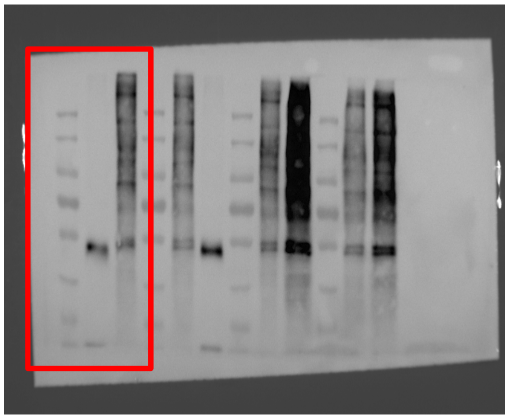

Ub

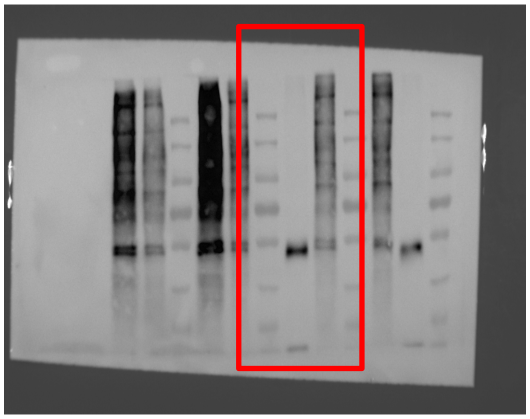

Ub

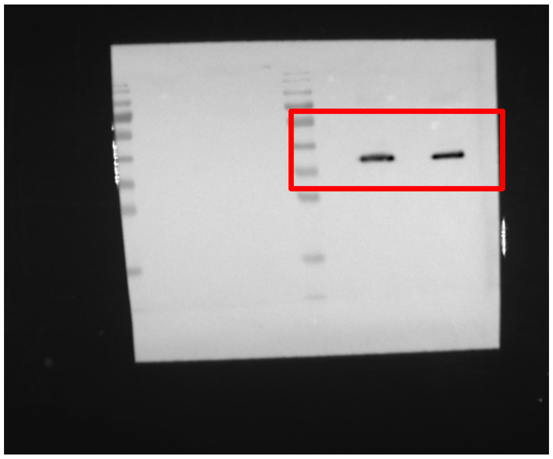

Myc

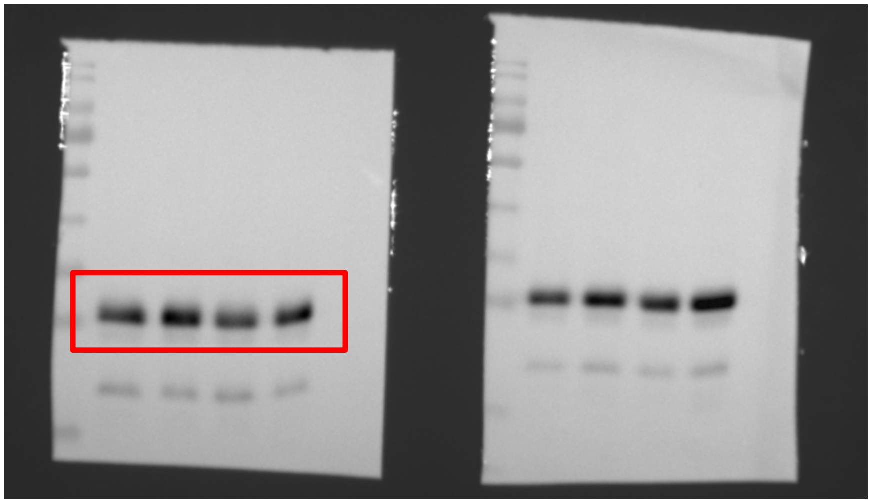

Flag

Fig 4A MCF7 cells

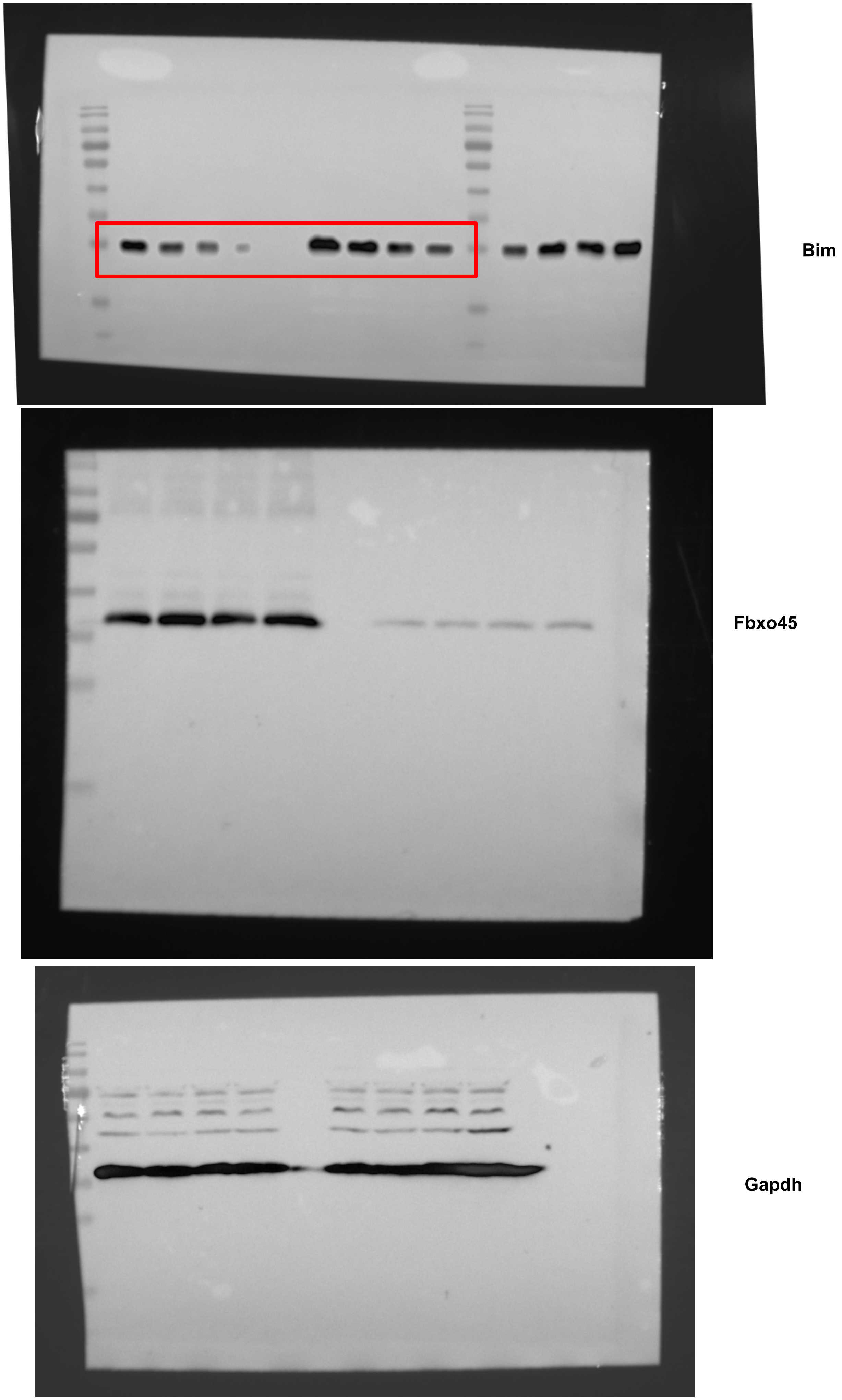

Fig 4A

MDM MB231 cells

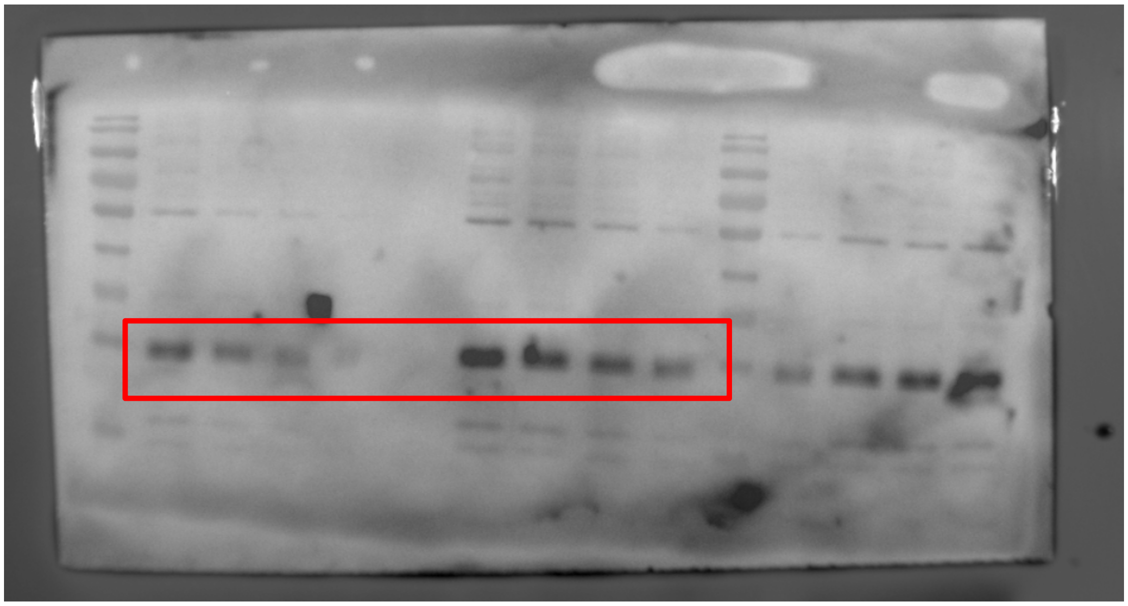

Bim

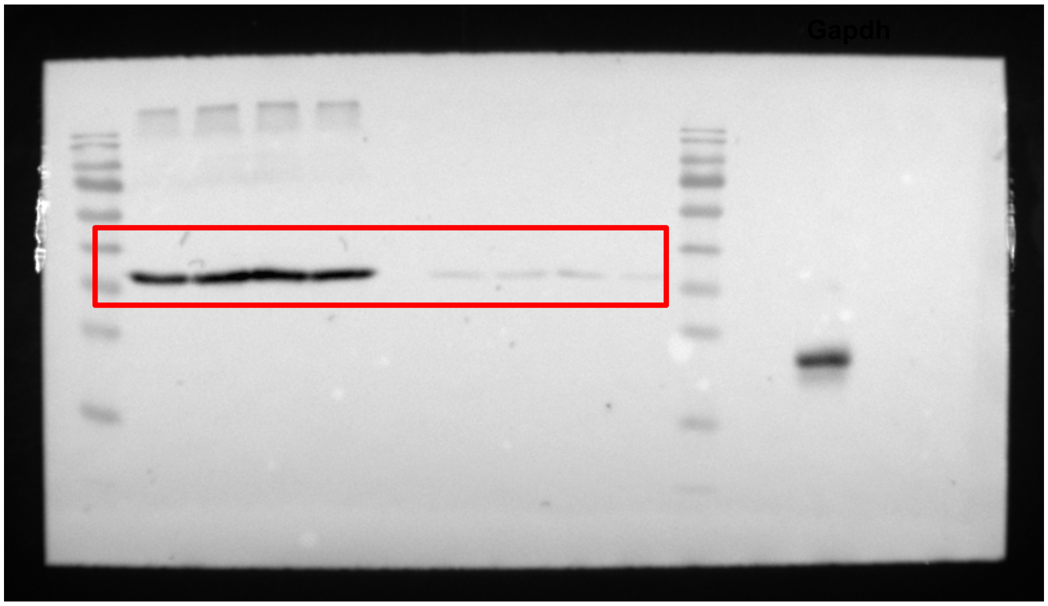

Fbxo45

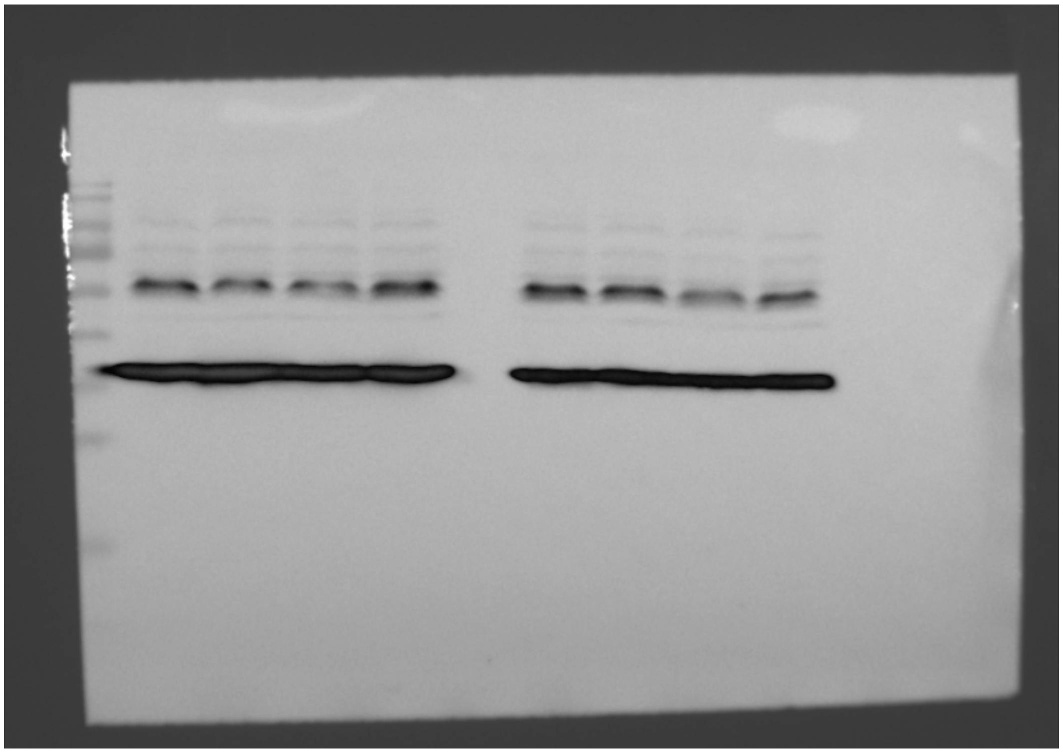

Gapdh

Fig 4C

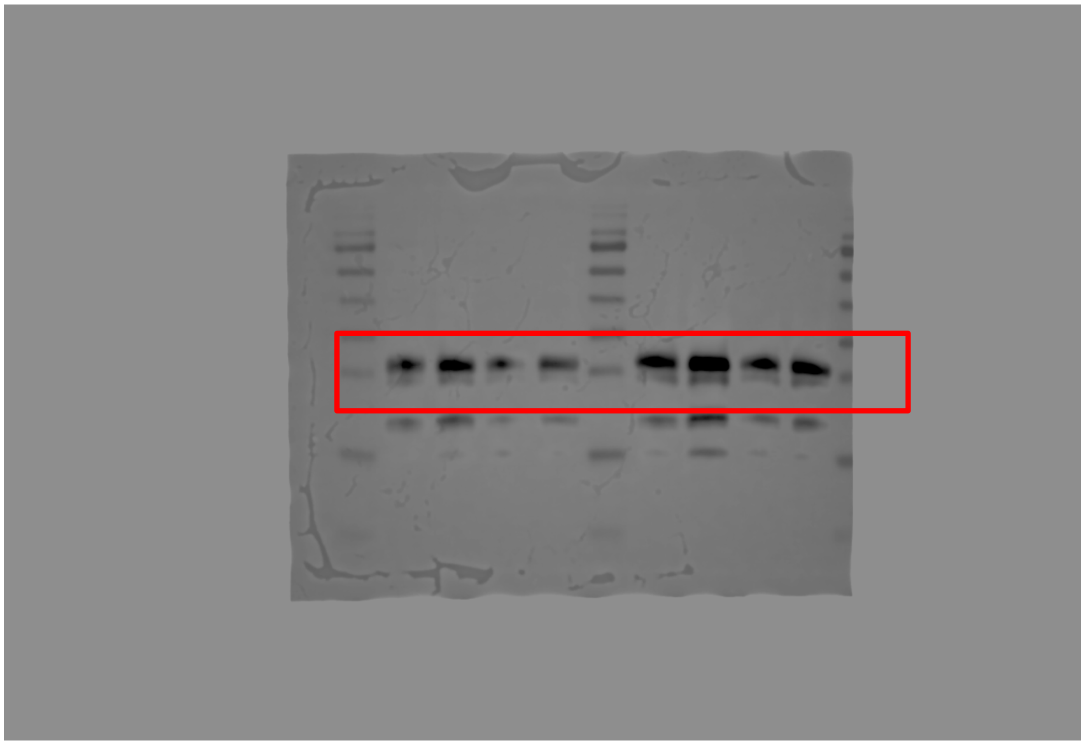

Bim

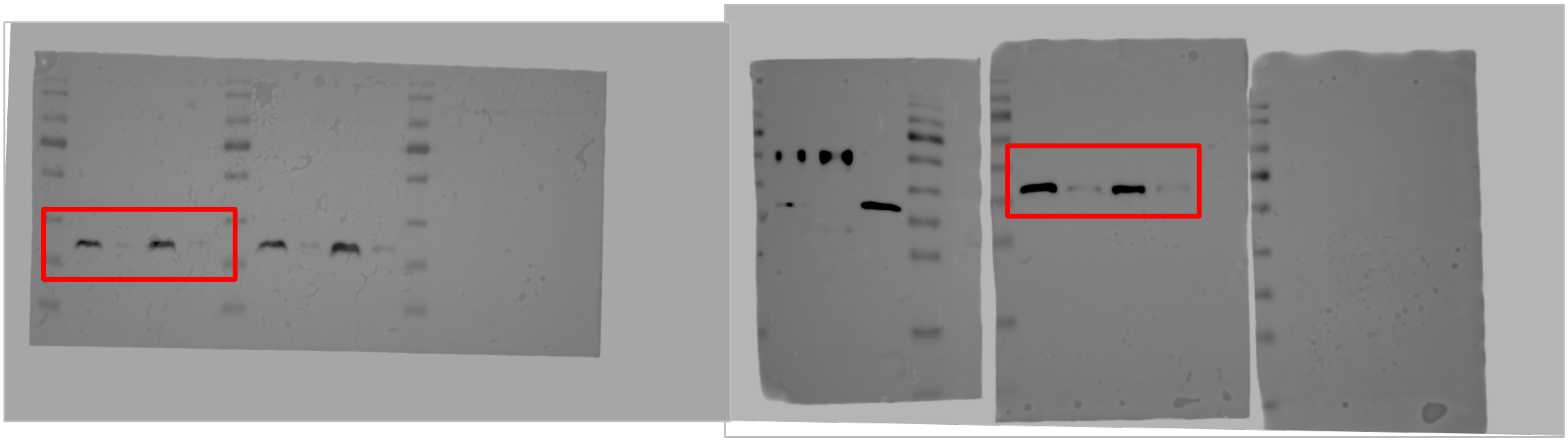

Fbxo45

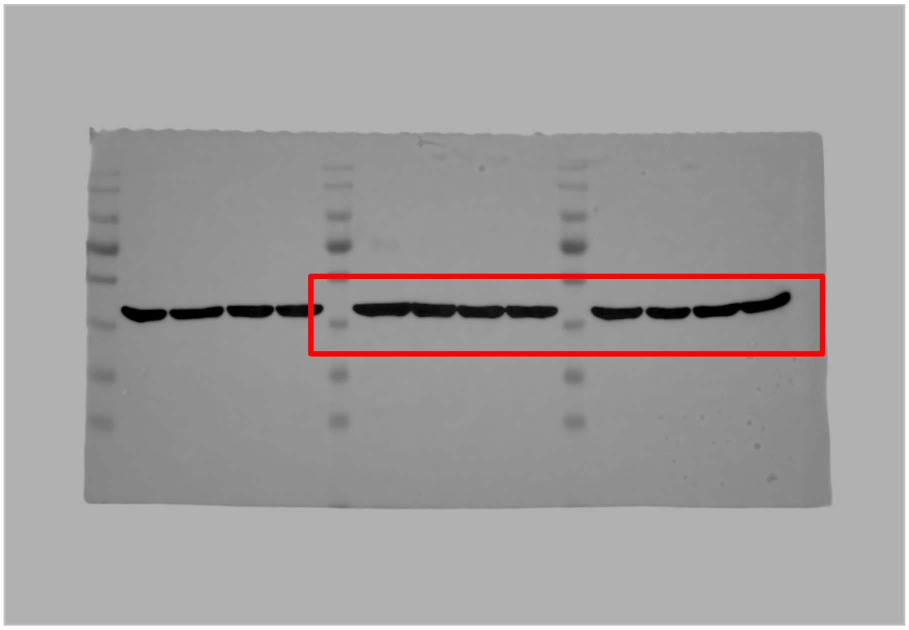

Actin

Fig 6D

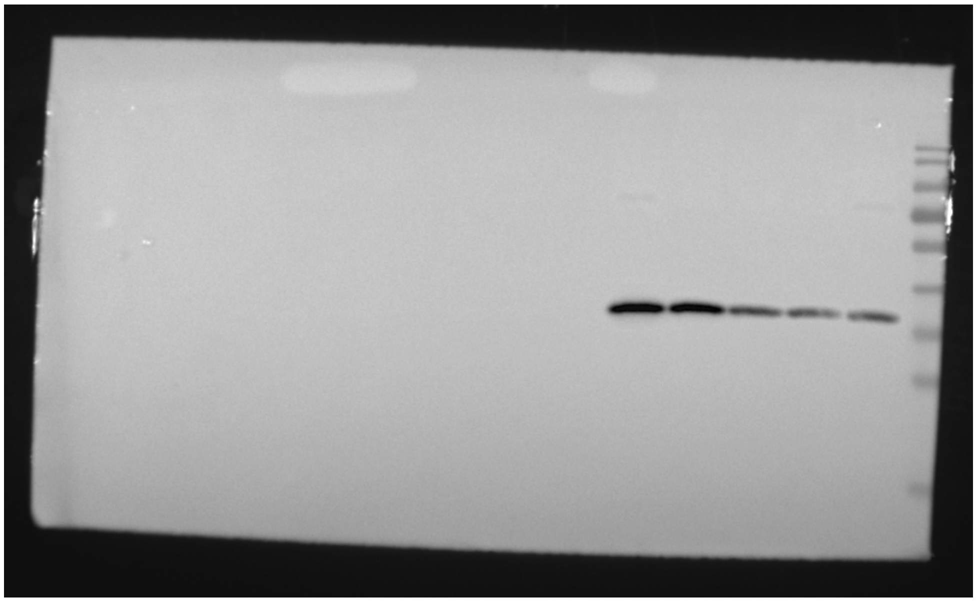

Flag

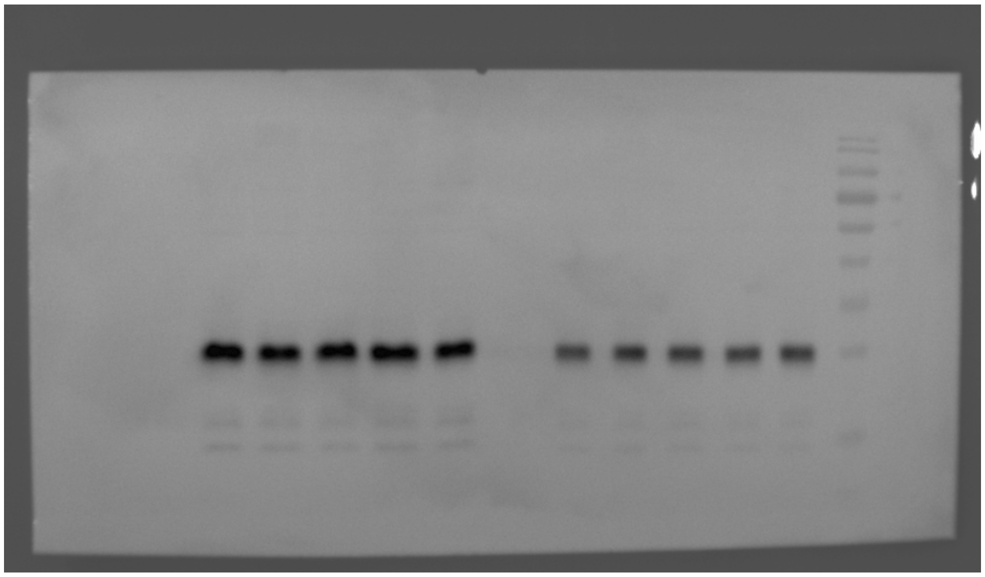

Bim

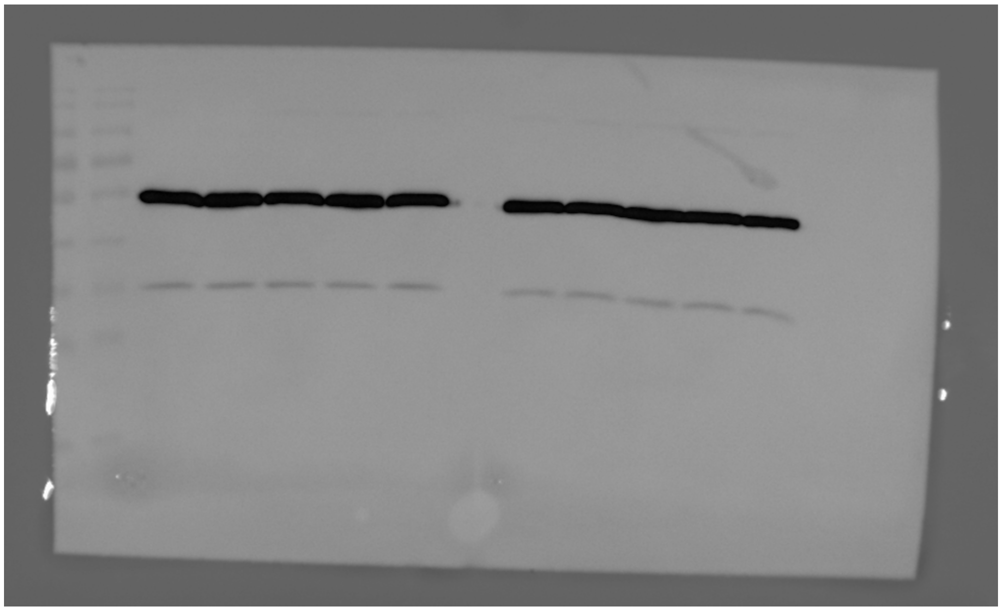

Tubulin

Supplementary figure 2F

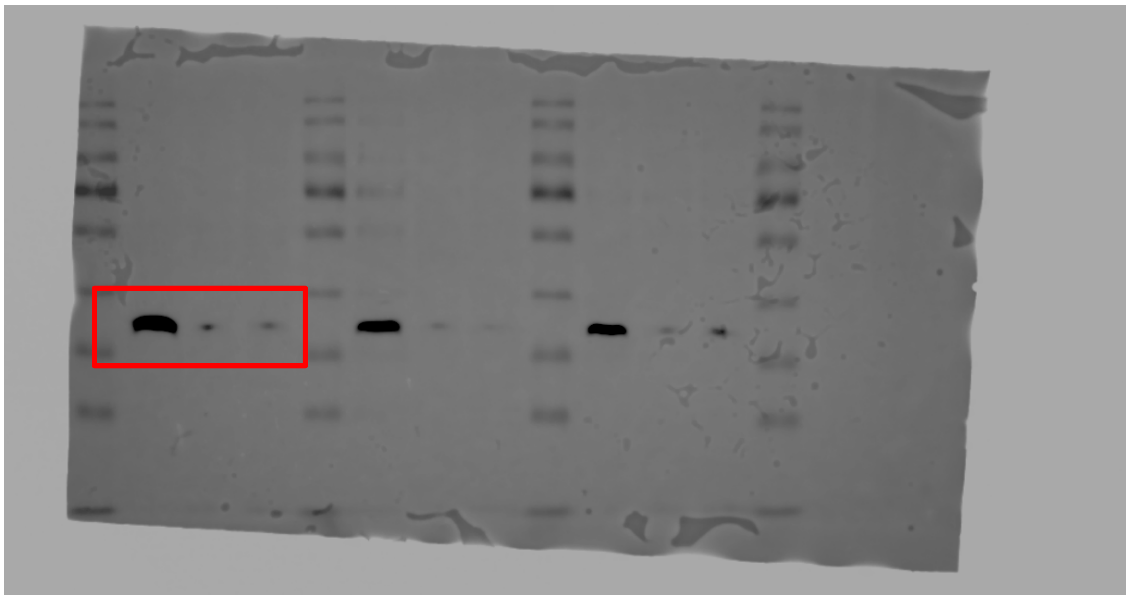

Fbxo45

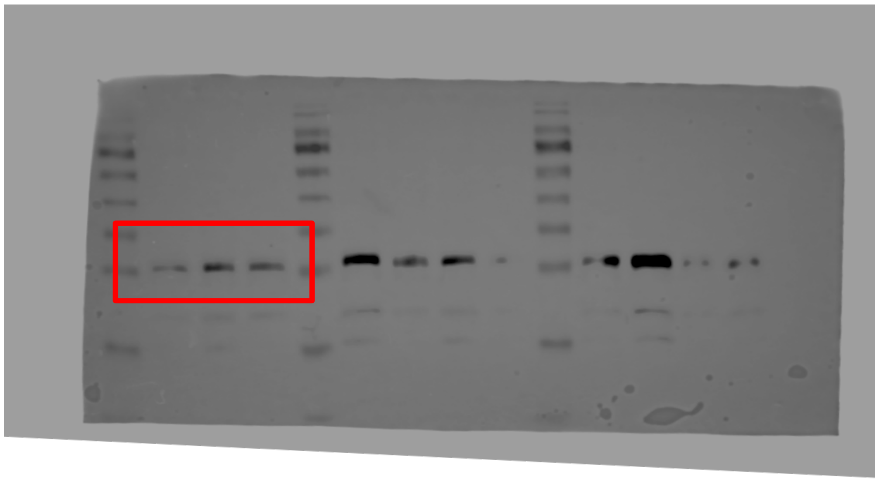

Bim

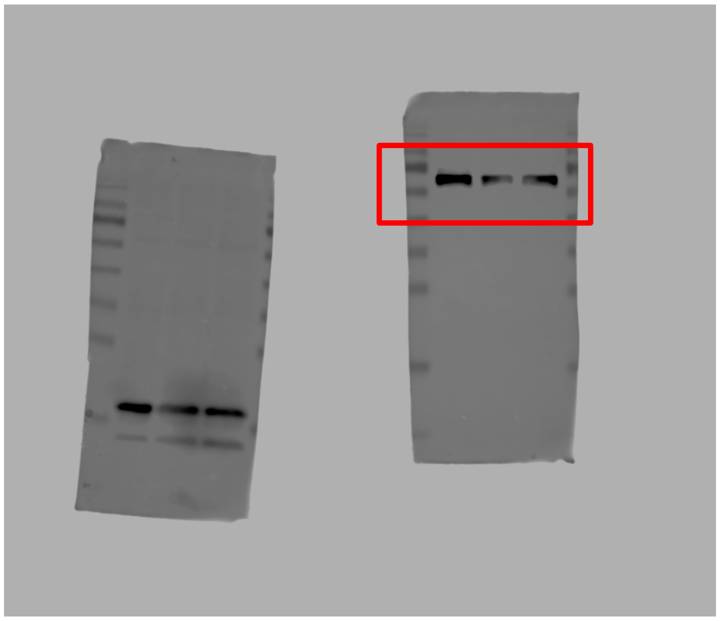

P62

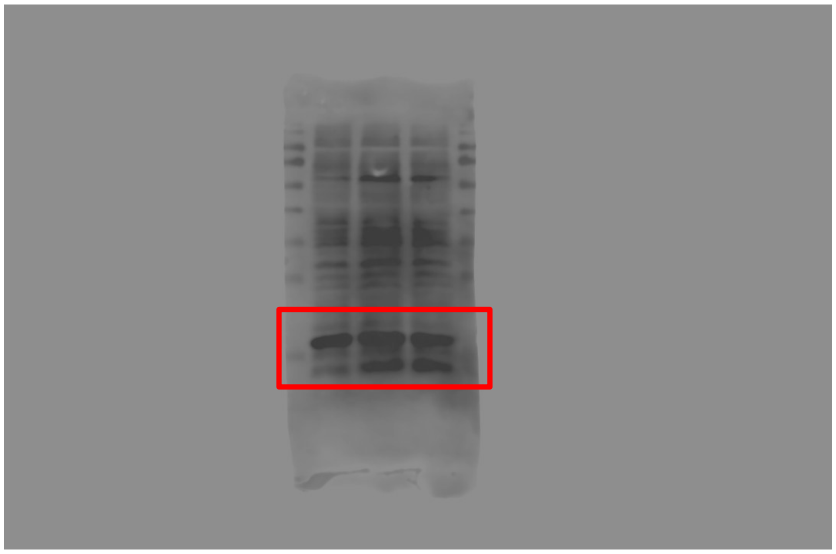

LC3

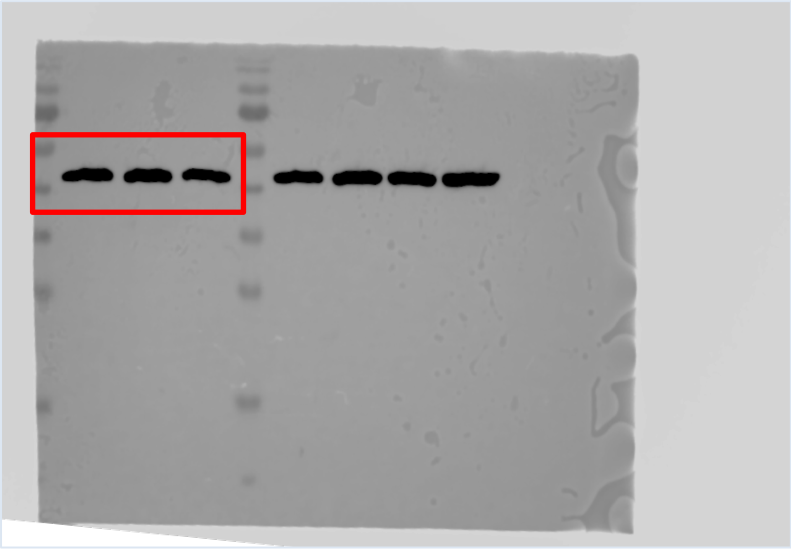

Actin
